# Establishment of Leptin-Responsive Cell Lines from Adult Mouse Hypothalamus

**DOI:** 10.1371/journal.pone.0148639

**Published:** 2016-02-05

**Authors:** Hiroshi Iwakura, Katsuko Dote, Mika Bando, Hiroyuki Koyama, Kiminori Hosoda, Kenji Kangawa, Kazuwa Nakao

**Affiliations:** 1 Medical Innovation Center, Kyoto University Graduate School of Medicine, Kyoto, Japan; 2 Department of Diabetes and Clinical Nutrition, Kyoto University Graduate School of Medicine, Kyoto, Japan; 3 Department of Human Health Sciences, Kyoto University Graduate School of Medicine, Kyoto, Japan; 4 National Cerebral and Cardiovascular Center Research Institute, Osaka, Japan; University of Rouen, France, FRANCE

## Abstract

Leptin resistance is considered to be the primary cause of obesity. However, the cause of leptin resistance remains incompletely understood, and there is currently no cure for the leptin-resistant state. In order to identify novel drug-target molecules that could overcome leptin resistance, it would be useful to develop *in vitro* assay systems for evaluating leptin resistance. In this study, we established immortalized adult mouse hypothalamus—derived cell lines, termed adult mouse hypothalamus (AMH) cells, by developing transgenic mice in which SV40 Tag was overexpressed in chromogranin A—positive cells in a tamoxifen-dependent manner. In order to obtain leptin-responsive clones, we selected clones based on the phosphorylation levels of STAT3 induced by leptin. The selected clones were fairly responsive to leptin in terms of STAT3, ERK, and Akt phosphorylation and induction of c-Fos mRNA induction. Pretreatment with leptin, insulin, and palmitate attenuated the c-Fos mRNA response to leptin, suggesting that certain aspects of leptin resistance might be reconstituted in this cellular model. These cell lines are useful tools for understanding the molecular nature of the signal disturbance in the leptin-resistant state and for identifying potential target molecules for drugs that relieve leptin resistance, although they have drawbacks including de-differentiated nature and lack of long-time stability.

## Introduction

The obesity pandemic is a predominant source of health problems worldwide, especially in the developed countries. Obesity causes diseases such as diabetes, dyslipidemia, hypertension, cardiovascular diseases, osteoarthritis, and cancer, resulting in increased morbidity and mortality. Current therapeutic options include dieting, exercise, cognitive behavioral therapy, anti-obesity drugs, and bariatric surgery. These therapies have some beneficial effects on weight reduction, but with the exception of bariatric surgery, their effects are often limited and short-term. Several types of anti-obesity drugs are commercially available, including lipase inhibitors, dopamine and noradrenaline reuptake inhibitors, anti-convulsants, and serotonin receptor antagonists [1]. However, the limited efficacy and adverse effects (sometimes very severe, e.g., valvulopathy for fenfluramine and suicidal tendencies for rimonabant) prevent the widespread usage of these drugs.

Leptin is a fat-derived hormone that plays a fundamental role in the regulation of food intake and energy homeostasis [2]. Mutations in leptin or leptin receptor genes result in severe obesity phenotypes in both humans [3, 4] and rodents [2, 5]. Exogenous administration of leptin to *ob*/*ob* or wild-type mice results in reductions in food intake and body weight [6–8]. Circulating leptin levels correlate with body fat mass [9–11], and are high in obesity. High circulating leptin levels and attenuated anorexic responses to exogenous leptin suggest that leptin resistance plays a role in obesity [11–13]. Hence, leptin resistance is considered to be one of the central causes of obesity [11, 14], and a great deal of effort has been expended on understanding the nature of the state. Although various hypotheses have been proposed [15], the nature of leptin resistance is not yet fully understood, and no currently available therapeutic drugs relieve leptin resistance.

In order to understand the signaling disturbance underlying the leptin-resistant state and identify novel drug-target molecules, it would be useful to develop *in vitro* assay systems for evaluating leptin resistance. In the current study, we developed immortalized adult mouse hypothalamus—derived cell lines with moderate responsiveness to leptin; these cells could be used in the development of cell-based assay systems to evaluate leptin resistance.

## Materials and Methods

### Generation of CgA-CreERT2 and CAG-LSL-SV40Tag Transgenic mice

**Fig 1:**
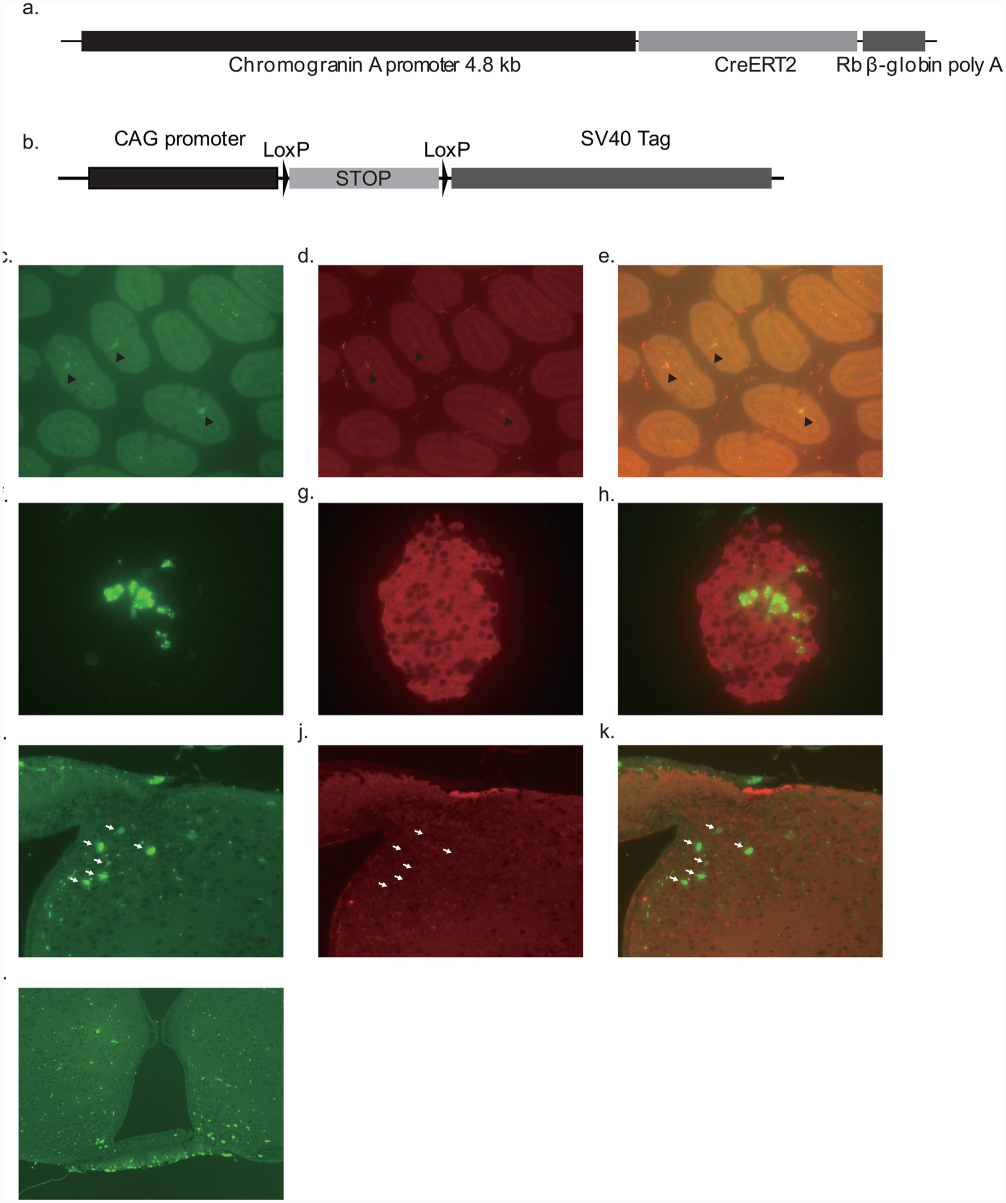
Establishment of hypothalamus-derived cell lines from chromogranin A promoter-CreERT2/CAG-promoter-lox-STOP-lox-SV40 Tag transgenic mice. a, b. DNA constructs for chromogranin A promoter-CreERT2 (a) and CAG-promoter-lox-STOP-lox-SV40 Tag transgenic mice (b). Cre-mediated recombination was confirmed by crossing chromogranin A promoter-CreERT2 mice with Cre-reporter mice (Rosa-CAG-LSL-ZsGreen1). c-k. Duodenum (c-e), pancreas (f-h) and hypothalamus (i-k) were stained with anti-chromogranin A (c: ZsGreen, d: chromogranin A, e: merge), anti-insulin (f: ZsGreen, g: insulin, h: merge), and anti-neuron specific enolase (NSE; i: ZsGreen, j: NSE, k: merge), respectively. l: Unstained hypothalamus.

We designed two types of fusion genes: one consisting of the chromogranin A promoter and CreERT2 (CgA-CreERT2), and the other consisting of the CAG-promoter [16], the floxed-stop sequence, and the SV40 T-antigen (CAG-LSL-SV40Tag) (Fig 1a and 1b). The purified fragments (10 μg/ml) were microinjected into the pronuclei of fertilized C57/B6N mouse (SLC, Shizuoka, Japan) eggs. Viable eggs were transferred into the oviducts of pseudopregnant female ICR mice (SLC) using standard techniques. Transgenic founder mice were identified by PCR. For experiments, we used heterozygous transgenic mice. Rosa-CAG-LSL-ZsGreen1 mice were obtained from Jackson Laboratory (Bar Harbor, ME, USA). Animals were maintained in a specific pathogen free facility on a 12-h light/12-h dark cycle at 25°C with free access to water and standard diet (SD; CE-2, 352 kcal/100 g, CLEA Japan, Tokyo, Japan). Animals were euthanized by cervical dislocation. All experimental procedures were approved by the Kyoto University Graduate School of Medicine Committee on Animal Research.

### Confirmation of Cre-mediated recombination of CgA-CreERT2 mice

CgA-CreERT2 mice were crossed with Rosa-CAG-LSL-ZsGreen1 Cre-reporter mice. The offspring were intraperitoneally injected with tamoxifen (1 mg/body) dissolved in corn oil once daily for 3 days. The duodenum, pancreas, and brain were resected and fixed with 10% formalin. Paraffin-embedded tissue sections were immunostained with anti-chromogranin A, anti-insulin, and anti-NSE (DAKO, Glostrup, Denmark) as described [17]. The sections were visualized with fluorescently (Alexa 555) labeled secondary antibodies (Life Technologies) on a BZ9000 microscope (KEYENCE, Tokyo, Japan).

### Cell culture

Hypothalamus resected from a CgA-CreERT2/CAG-LSL-SV40Tag double-transgenic mouse was minced and digested with 0.25% trypsin-EDTA (Gibco, Carlsbad, CA, USA) at 37°C for 20 minutes. After washing with phosphate-buffered saline (PBS), cells were cultured in DMEM (11995–065, Gibco) supplemented with 10% FBS, B-27 supplement, and 1 μM tamoxifen at 37°C in 10% CO_2_ on a feeder layer of mitomycin C—treated mouse embryonic fibroblasts. After 3 days of tamoxifen treatment, the media was replaced with DMEM supplemented with 10% FBS, B-27 supplement, 100 U/ml penicillin, and 100 μg/ml streptomycin. After 2–3 weeks of culture, colonies were picked onto poly D-lysine—coated 96-well plates and cultured in DMEM supplemented with 10% FBS, 100 U/ml penicillin, and 100 μg/ml streptomycin until the cell number was sufficient for mRNA evaluation. After mRNA evaluation, cells from the selected colony were cloned by the dilution method onto a feeder layer of mitomycin C—treated embryonic fibroblasts in 96-well plates. Established cell lines were cultured in DMEM supplemented with supplemented with 10% FBS, 100 U/ml penicillin, and 100 μg/ml streptomycin on standard culture plate. Established cell lines were frozen stocked at early passage. We used the cells at the passage number below 20 after the initiation of culture from the frozen stocks to avoid the phenotypic changes.

### Electron microscope

Electron microscope study was performed as described previously [18].

### Immunocytochemistry

Cells cultured in 24 well plate were fixed with 10% formalin for 15 min and permeabilized with 0.1% Triton X-100 in PBS. Cells were incubated with anti-AgRP (1:10 at final dilution) (R&D systems, Minneapolis, MN, USA), and anti-αMSH (1:20) (Phoenix Pharmaceuticals, Inc., Burlingame, CA, USA), then visualized with fluorescently (Alexa 488 and 555) labeled secondary antibodies (Life Technologies) on a BZ9000 microscope (KEYENCE).

### Reverse phase HPLC and tricine SDS-PAGE

Medium were collected after 48 hours culture of the cells. After centrifugation, the medium were applied to Sep-Pak C18 cartridges (Waters Corp., Milford, MA) pre-equilibrated with 0.9% saline. Cartridges were washed in saline and 5% CH3CN/0.1% trifluoroacetic acid (TFA) and eluted with 60% CH_3_CN/0.1% TFA. Eluates were lyophilized and reconstitute with Tricine SDS sample buffer with reducing agent (Life Technologies). Tricine SDS-PAGE and Western blot analysis were performed as described previously [19] using anti-AgRP antibody (1:2000).

### Leptin treatment

Two days before the assay, cells were seeded on 12-well plates in DMEM supplemented with 10% FBS at 37°C in 10% CO_2_. Sixteen hours before the assay, the medium was replaced with DMEM containing 0.5% BSA and other supplements as indicated (leptin, insulin, or palmitate). Leptin (100 nM, Peprotech, Rocky Hill, NJ, USA) was added before collection of the cells for quantitative RT-PCR or Western blot analysis.

### Quantitative RT-PCR

Quantitative RT-PCR was conducted as described previously [20]. Primer and TaqMan probe sequences are presented in S1 Table. mRNA expression of each gene was normalized to the levels of 18S rRNA.

### Intracellular Ca^2+^ measurement

Intracellular Ca^2+^ levels ([Ca^2+^]_i_) were measured with Calcium Kit II—Fluo 4 (Dojindo, Kumamoto Japan) and FDSS/μcell (Hamamatsu Photonics, Shizuoka, Japan). Neuropeptide (NPY), vasopressin (AVP), thyrotropin-releasing hormone (TRH), peptide YY, cholecystokinin (CCK), neuromedin S, neuromedin U, orexin B, calcitonin, ghrelin, and luteinizing hormone releasing hormone (LH-RH) were obtained from Peptide Institute (Osaka, Japan). Pancreatic polypeptide (PP), neurotensin, muscarine, adrenaline, histamine, serotonin, melatonin, glutamate, methoxyamine, and clonidine were purchased from Sigma-Aldrich Japan (Tokyo, Japan).

### Intracellular cAMP measurement

Intracellular cAMP levels were measured with cAMP assay kit and 2104 Envision multilabel reader (Perkin Elmer, Waltham, MA). Acetylcholine, noradrenaline, GABA, somatostatin and forskolin were purchased from Sigma-Aldrich Japan. GIP, endothelin and oxytocin were obtained from Peptide Institute.

### Immunoblot analysis

Cells were washed once in PBS and lysed in 1 ml lysis buffer (40 mM HEPES, 10 mM EDTA, 100 mM NaF, 10 mM sodium pyrophosphate, 1 mM Na_3_VO_4_, 0.1 mg/ml aprotinin, 1 mM phenylmethylsulfonyl fluoride, 50 μM okadaic acid, and 1% Nonidet, pH 7.5). Protein concentrations were measured using the Pierce BCA Protein Assay Kit (Thermo Scientific, Rockford, IL, USA). Each sample was reduced by adding NuPAGE LDS Sample Buffer (Invitrogen, Carlsbad, CA, USA) and Bolt Sample Reducing Agent (Invitrogen). The treated samples were subjected to Bolt Bis-Tris gels and electrophoretically transferred to polyvinylidene fluoride membranes (Invitrogen). Transferred membranes were blocked with ECL Blocking Agent (GE Healthcare, Buckinghamshire, UK) and then incubated with anti-STAT3 (1:2000), anti-phospho-STAT3 (1:2000), anti-ERK (1:1000), anti-phospho-ERK (1:2000), anti-Akt (1:1000) and anti-phospho-Akt antibodies (Cell Signaling, Danvers, MA). After washing with PBS–0.1% Tween-20, membranes were incubated with secondary antibodies and developed with SuperSignal West Femto Maximum Sensitivity Substrate (Thermo Fisher Scientific, Waltham, MA, USA). Signal was detected with a Lumino-Image Analyzer LAS-3000 Mini System (Fuji Photo Film, Tokyo, Japan).

### Small interfering RNA (siRNA)

Synthetic siRNAs and a negative control were purchased from Invitrogen (Carlsbad, CA). Two types of siRNAs specific for TLR4 were used: CUAUCUAGAUCUUAGUAGA (si1) and CUCCAUAGACUUCAAUUAU (si2). siRNAs were delivered into cells using Lipofectamine RNAi Max (Invitrogen).

### Statistical analysis

All values are expressed as means ± SE. The statistical significance of differences in mean values was assessed by ANOVA using a post-hoc test (Tukey’s test) or Student’s *t*-test, as appropriate. *p* < 0.05 was considered to indicate a statistically significant difference. Statistical analysis was performed using Statcel 2 (OMS, Saitama, Japan). All the experiments were conducted at least twice to confirm the reproducibility.

## Results

In order to immortalize adult hypothalamic neurons, we used tamoxifen-inducible Cre/Lox technology with a floxed stop cassette. Two types of transgenic mice, chromogranin A promoter-CreERT2 (CgA-CreERT2) and CAG-promoter-lox-STOP-lox-SV40 Tag (CAG-LSL-Tag) transgenic (Tg) mice were created using the DNA constructs, as presented in Fig 1a and 1b. Theoretically, SV40-Tag expression can be induced in chromogranin A—expressing cells (endocrine cells or neurons) by tamoxifen administration to CgA-CreERT2/CAG-LSL-Tag double transgenic mice, resulting in immortalization of the target cells. When we crossed CgA-CreERT2 Tg mice with Cre-reporter mice (Rosa-CAG-LSL-ZsGreen1) [21], bright fluorescence was observed in chromogranin A-positive enteroendocrine cells (Fig 1c–1e) and in insulin-positive pancreatic endocrine cells (Fig 1f–1h) after intraperitoneal tamoxifen administration. In the brain, fluorescence was detected in the neuron specific enolase (NSE) positive cells (Fig 1i, 1j and 1k) in the median eminence and in the arcuate nucleus of mediobasal hypothalamus (Fig 1l), indicating that CreERT2 functions in neural cells following tamoxifen administration. Fluorescence was barely detectable in other brain areas (data not shown), probably due to the limited permeability of tamoxifen across the blood—brain barrier. We then used tamoxifen treatment to transform hypothalamic cells from a CgA-CreERT2/CAG-LSL-Tag double-transgenic mouse *in vitro*. Specifically, the hypothalamus was resected from CgA-CreERT2/CAG-LSL-Tag double-transgenic mice, minced, and incubated with tamoxifen-containing medium. After 2 weeks of culture, several dozen colonies arose, and these were picked and cultured separately. Levels of mRNAs encoding neural markers, neural peptides, and receptors were assessed in the viable colonies (S2 Table). These colonies expressed high levels of SV40 Tag mRNA (data not shown), indicating that recombination occurred successfully following tamoxifen treatment *in vitro*. We selected colony 11 for further experiments because of its relatively high expression of hypothalamic neural peptides. We carried out dilution cloning of this colony on mitomycin C—treated mouse embryonic fibroblasts, and ultimately obtained 47 clones of hypothalamus-derived immortalized cells. We designated the established cell line as adult mouse hypothalamus (AMH) cells.

**Fig 2:**
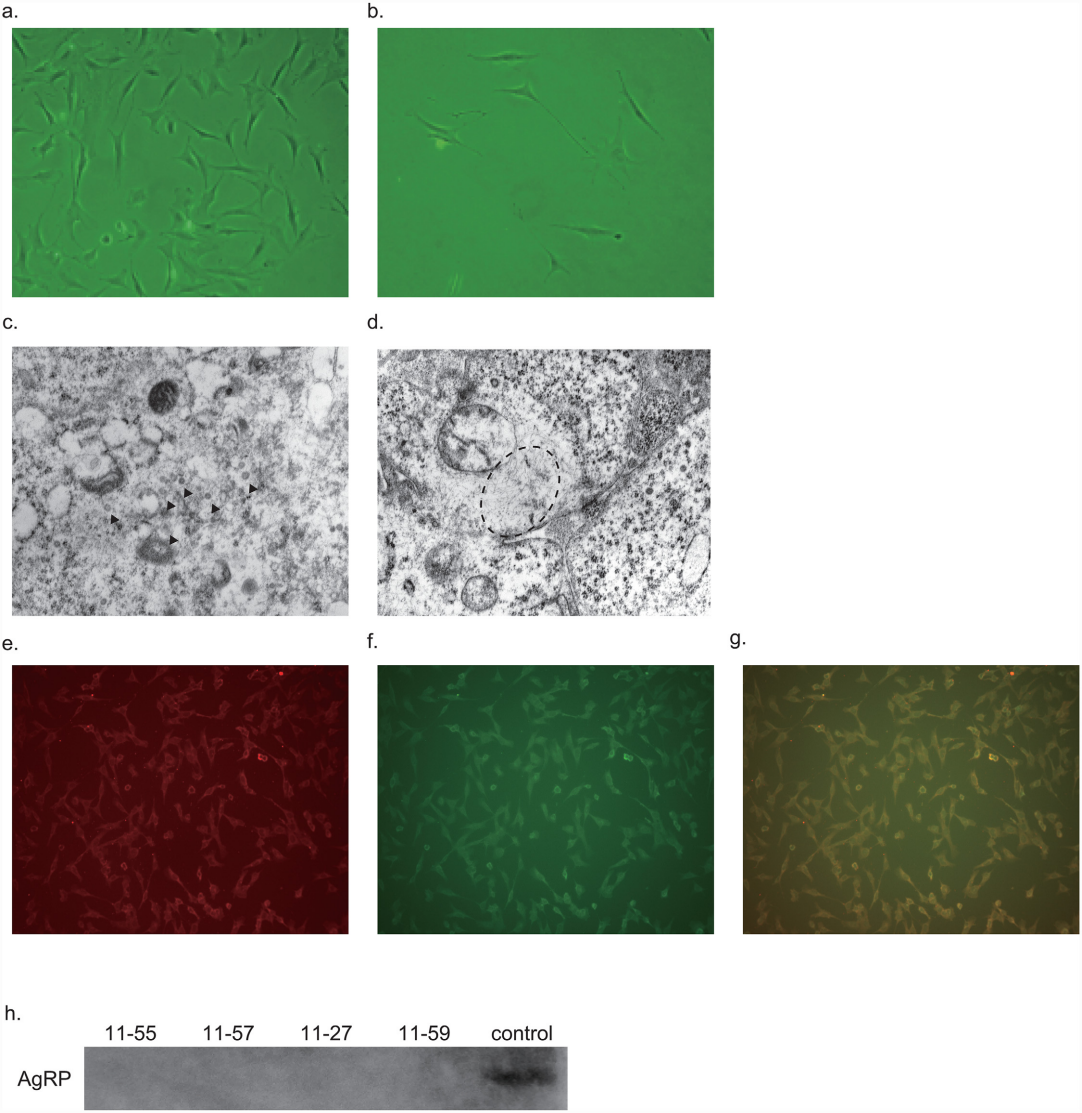
Morphology of established hypothalamus-derived cell lines. a, b. Representative images of established hypothalamus-derived cell lines. c, d. Representative images of electron microscopy. A few secretory granules (c: arrow) and intermediate filaments (d: dotted circle) were observed. e-g. Cells (11–55) were stained with anti-AgRP and anti αMSH antibodies (e: AgRP, f: αMSH, g: merge). h. Western blot analysis after Tricine SDS page of the culture medium of the cell lines and AgRP control peptide (50 pmol) with anti-AgRP antibody.

AMH cells grow well in usual culture plate (the doubling time was about 24 hours). AMH cells exhibited a fibroblast-like morphology under normal culture conditions (Fig 2a). In very sparse culture, some cells extend neurite-like structures (Fig 2b). These clones expressed mRNAs encoding neural peptides, neural markers, and leptin receptor (S3 Table). Although we found a few vesicle-like structures or intermediate filaments in the cells (Fig 2c and 2d) by electron microscope, no synapse formation was observed. Furthermore, we failed to detect membrane potential changes by neurotransmitters using DiBAC_4_(3) (data not shown). These results suggest that some of the neural characteristics were lost during the immortalization process. In addition, the expression pattern of neural peptides mRNA in these cell lines were unusual combination (S3 Table). Double-label immunocytochemistry showed weak AgRP-like and αMSH-like immunoreactivities existed in the same cells (Fig 2e–2g), suggesting that these cell lines were de-differentiated in terms of neural peptides expression. We could not detect AgRP peptide in the culture medium of the established cell lines, probably due to the low expression levels of the peptide.

**Fig 3:**
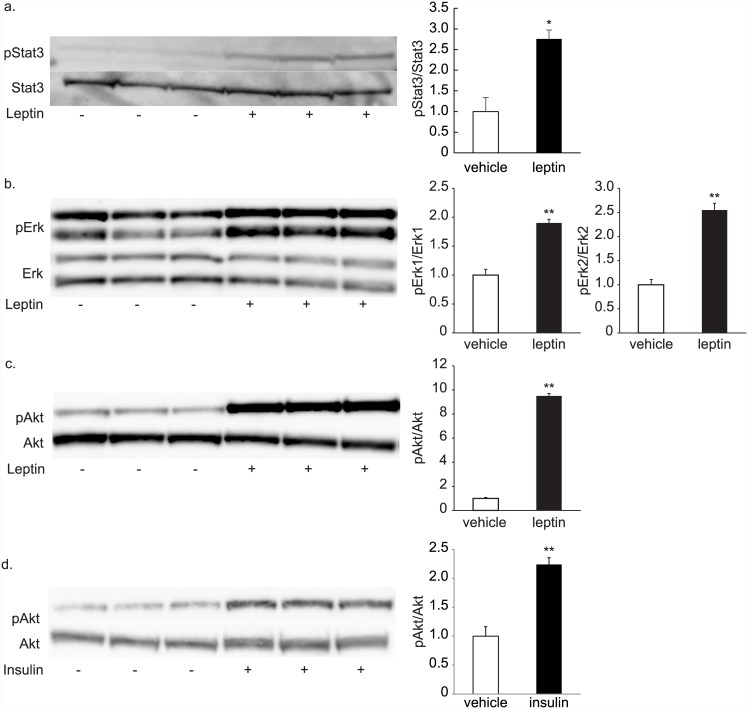
STAT3, ERK, and Akt phosphorylation induced by leptin treatment in the established hypothalamus-derived cell lines. a–c. Stat3 (a), Erk1, 2 (b), and Akt (c) phosphorylation was induced by addition of 100 nM leptin for 15 min in clone 55 of the hypothalamus-derived cell lines. d. Akt phosphorylation was induced by addition of 100 nM insulin for 5 min in the clone 55 of hypothalamus-derived cell lines.*: n = 3, *p* < 0.05, **: *p* < 0.01 compared to vehicle.

We evaluated the leptin responsiveness of each clone by assessing the induction of Stat3 phosphorylation in response to leptin treatment (S1 Fig). Among 47 clones, we selected several clones for their relatively high Stat3 phosphorylation rates. These clones exhibited significant elevation of Stat3 phosphorylation in response to leptin treatment, relative to vehicle treatment (Fig 3a: representative clone AMH11-55, S1 Fig). Leptin also caused phosphorylation of ERK1/2 and Akt in these clones (Fig 3b and 3c, S1 Fig), indicating that major intracellular signaling pathways evoked by leptin, including the JAK-Stat, MAPK, and PI3K pathways, were preserved in these clones. Moreover, insulin also stimulated phosphorylation of Akt in these cells (Fig 3d).

**Fig 4:**
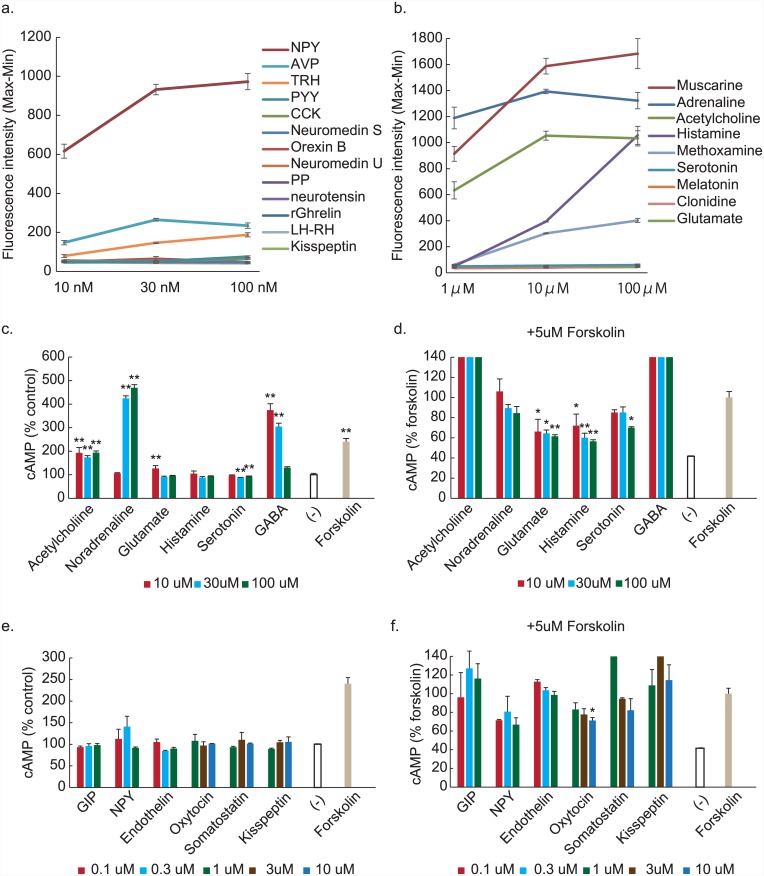
Increased intracellular Ca2^+^ and cAMP concentration following addition of known peptide hormones or neurotransmitters. a, b. NPY (a), muscarine, adrenaline (or the α1 agonist methoxamine, but not the α2 agonist clonidine), and histamine (b) evoked profound elevation of [Ca^2+^]_i_. AVP and TRH induced [Ca^2+^]_i_ elevation to a lesser extent (a). Data from clone 11–55 is presented as the difference between maximum and minimum fluorescence intensities (max—min). Detailed fluorescent curves are presented in S2 and S3 Figs. c-f. Acetylcholine, noradrenaline and GABA elevated intracellular cAMP (c), while glutamate, histamine and serotonin suppressed it when added with 5μM forskolin (d). Oxytocin also suppressed cAMP (f). n = 3, **: *p* < 0.01, **: *p* < 0.05 relative to control.

**Fig 5:**
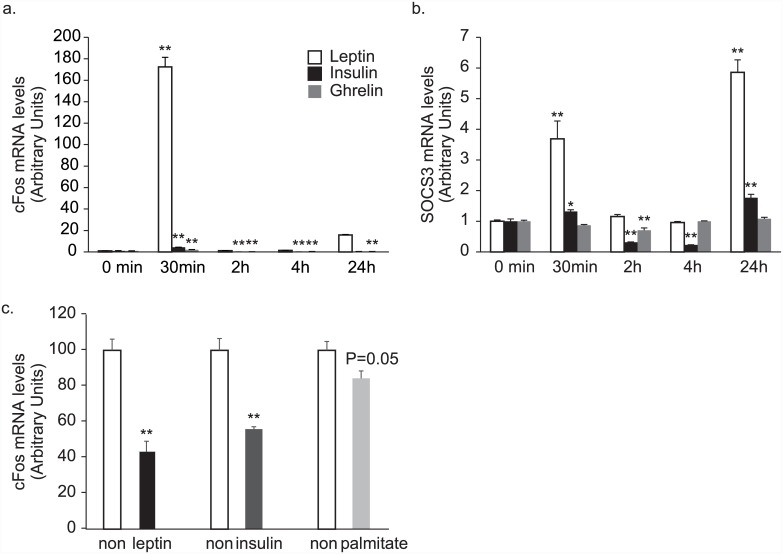
c-Fos mRNA responses to leptin. c-Fos (a) and SOCS3 (b) mRNA responses of clone 11–55 to the addition of 100 nM leptin, insulin, or ghrelin to the incubation medium. *: *p* < 0.05, **: *p* < 0.01 relative to 0 min. n = 4. c. c-Fos mRNA responses 30 min after the addition of 100 nM leptin to clone 11–55 pretreated with leptin (10 nM), insulin (100 nM), or palmitate (200 μM) for overnight. **: *p* < 0.01 relative to a non-pretreated sample. n = 4.

Next, we examined the responses of AMH11-55 cells to known peptide hormones or neurotransmitters. We found that NPY, muscarine, adrenaline (or the α1 agonist methoxamine, but not the α2 agonist clonidine), and histamine evoked profound elevation of [Ca^2+^]_i_ levels (Fig 4a and 4b; S2 and S3 Figs). AVP and TRH also induced [Ca^2+^]_i_ elevation to a lesser extent (Fig 4a; S2 Fig). Furthermore, acetylcholine, noradrenaline and GABA elevated intracellular cAMP levels, while glutamate, histamine, serotonin, and oxytocin suppressed them when added with forskolin (Fig 4c–4f). Another clone AMH11-57 also responded to known neurotransmitters (acetylcholine, noradrenaline, glutamate, histamine, and GABA), although the response pattern was slightly different from that of clone 11–55 (S4 Fig).

c-Fos mRNA levels are elevated during neural activation [22]. Addition of leptin significantly stimulated c-Fos mRNA expression in AMH11-55 cells and in other clones (Fig 5a, S5 Fig), with concomitant elevation of Socs 3 mRNA levels (Fig 5b). NPY and POMC mRNA levels, however, were not changed with leptin treatment as expected. Leptin did not affect these mRNA levels in clone 55 and clone 59, and rather upregulated NPY mRNA at 6 and 8 hours in clone 57 (S6 Fig). Since leptin should downregulate NPY and upregulate POMC in feeding-related neurons [14], these responses were not the expected ones. Insulin and ghrelin also induced small but significant elevation of cFos mRNA expression (Fig 5a). We then examined if some of the characteristic features of serum in obesity (high leptin, high insulin, and high free-fatty acids) affect leptin responsiveness. We used palmitate for fatty acids, since palmitate is one of the major components in the serum free fatty acids and has been suggested to induce leptin resistance [23] as well as insulin resistance in vivo [24]. Pretreatment with leptin or insulin significantly attenuated (and pretreatment with palmitate tended to attenuate) leptin-induced c-Fos expression in these cells (Fig 5c).

**Fig 6:**
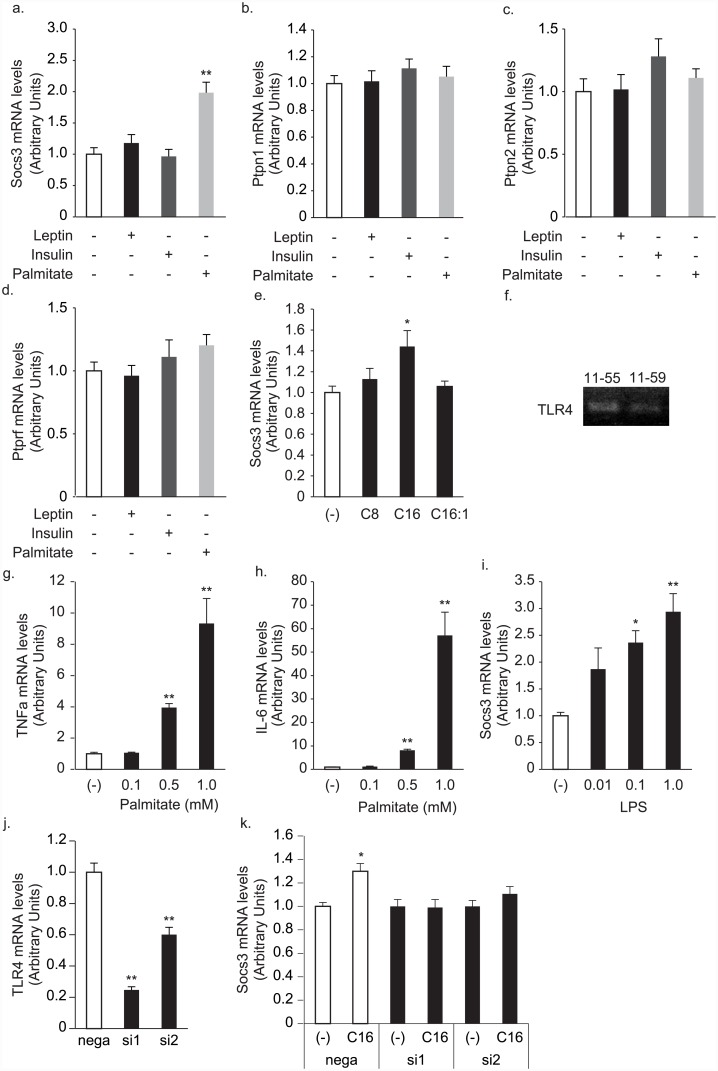
Socs3, Ptpn1, Ptpn2 and Ptprf mRNA levels by high leptin, insulin and palmitate containing medium. Socs3 (a), Ptpn1 (b), Ptpn2 (c) and Ptprf (d) mRNA levels of clone 55 after overnight incubation with 10 nM leptin, 100 nM insulin and 200 μM palmitate containing medium. **: *p* < 0.01 relative to a non-pretreated sample. n = 6. e. The effects of 1 mM octanoate (C8), palmitate (C16), and palmitoleate (C16:1) on Socs3 mRNA expression levels in clone 11–55 cells. f. TLR mRNA expressions in 11–55 and 11–59 cells. g, h. Palmitate significantly elevated TNFα and IL-6 mRNA levels in 11–55 cells. i. LPS significantly elevated Socs3 mRNA levels. *: *p* < 0.05, **: *p* < 0.01 relative to a non-pretreated sample. n = 6. j. k. Knockdown of TLR4 expression by siRNAs (j) abolished the palmitate-induced Socs3 mRNA elevation (k). *: *p* < 0.05, **: *p* < 0.01 relative to siRNA negative control. n = 6.

We finally examined the mRNA expression of known inhibitors of leptin or insulin signaling [15]; Socs3 [25–27], Ptprn1 [28–30], Ptprn2 [31] and Ptptrf [32] (Fig 6). Treatment of insulin, leptin did not induce these genes, whereas palmitate significantly elevated Socs3 mRNA levels in AMH11-55 cells (Fig 6a). Treatment of octanoate and palmitoleate did not induce Socs3 mRNA (Fig 6e), suggesting that only saturated long-chain fatty acids can induce Socs3. Saturated long-chain fatty acids can be recognized by Toll-like receptor4 [33]. Indeed, the cells expressed TLR4 mRNA (Fig 6f, S7 Fig). Palmitate induced TNFα and IL-6 mRNA (Fig 6g and 6h), which is regulated by TLR4 signaling pathway [33], while TLR4 ligand lipopolysaccharide induced Socs3 mRNA in the cells (Fig 6i). Knockdown of TLR4 by siRNAs abolished the Socs3 mRNA elevation induced by palmitate treatment (Fig 6j and 6k). These results suggested that palmitate elevated Socs3 mRNA by TLR4 receptor signaling pathway.

## Discussion

Earlier studies examining leptin signaling pathways used cell lines such as HEK293 or COS, forced to express leptin signaling molecules [34]. Although these cell models may have provided fundamental knowledge about leptin signaling pathways, transfected cell models have several limitations. In particular, forcibly expressed signaling molecules are often over-expressed to super-physiological levels due to the use of a strong promoter such as the CMV promoter. Thus, the observed signaling may not always represent the physiological events that occur in leptin target cells *in vivo*. Furthermore, because these cell lines are derived from non-neural tissues (e.g., HEK293 from human embryonic kidney, COS from Chinese hamster ovary), their expression patterns of receptors or signaling molecules may be very different from those in leptin-target cells (primarily hypothalamic neurons). These limitations may have hampered the development of useful cell-based models for assessing leptin resistance *in vitro*.

In the current study, we developed adult mice hypothalamus—derived cell lines, which exhibited moderate leptin responsiveness (phosphorylation of STAT3, ERK1/2, and Akt). Moreover, the cells also responded to other neurotransmitter or hormones involved in body energy homeostasis, such as insulin, NPY, adrenaline, histamine, or muscarine in addition to basic neurotransmitters; glutamate and GABA. However, we found no effects of leptin on NPY and POMC mRNA levels in clone 55 and 59, and found rather stimulatory effects on NPY mRNA in clone 57. These responses were not expected ones, since leptin usually downregulates NPY and upregulates POMC [14]. They may have originated in feeding-related area since they express NPY and POMC, but they did not react like feeding-related neurons.

The characteristics of hypothalamic neurons are divergent; consequently, these cells cannot be simply classified according to the neural peptides they express, and neurons expressing the same peptide do not always exhibit the same response to stimuli. For example, proopiomelanocortin (POMC) neurons are considered to be a major target of leptin. However, only 60% of POMC neurons in POMC-EGFP mice exhibit STAT3 phosphorylation in response to leptin administration [35]. Similarly, only 25% of POMC neurons in the ARC express c-Fos in response to leptin administration [36]. The origin of this diversity among neurons remains unknown. Our established cell lines presumably arose from single specific neurons with different characteristics, although they lost some of their original properties during the immortalization process, which is inevitable when using immortalized cells. Expression of unusual combination of neural peptides in the same cells and the lack of regulation of neural peptides expressions by leptin suggest de-differentiation of the cells. It should be noted that long time culture further impaired the phenotypes of the cells including leptin responsiveness eventually. The phenotypes are not stable over the long-time period, presumably due to the relatively high expression levels of SV40 Tag. Nonetheless, our established cell lines should be useful tools for certain purposes, such as analysis of intracellular signaling or development of screening assay systems.

Hypothalamus-derived cell lines were previously developed by other groups. Belsham *et al*. developed cell lines from embryonic [37] or adult mouse hypothalamus [38]. They used an SV40-Tag—expressing retrovirus, which can only infect dividing cells. Furthermore, they used ciliary neurotrophic factor (CNTF) to trigger neurogenesis in primary cultures of adult mouse hypothalamus, enabling the immortalization of adult neurons [38]. Thus, their methods allowed immortalizations of neurons that respond to CNTF; by contrast, in our approach, which uses the tamoxifen-dependent Cre-Lox system, theoretically all neurons could be immortalized. Therefore, AMH cells could be derived from different population of the neurons other than those immortalized in their studies. Considering the heterogeneity of the hypothalamic neurons, it would be reasonable to use different cell lines from different origin as many as possible, before proceeding to *in vivo* studies. Our cell lines would be one of those cells.

Some of the clones generated by Belsham et al. exhibited leptin responsiveness [37, 39, 40], as well as attenuation of the response in certain settings, such as pretreatment with leptin or insulin [41–43]. We obtained essentially the same results regarding leptin responsiveness and attenuation of the response by pretreatment with leptin and insulin. Besides, our results also suggest that palmitate directly interfere with the leptin signaling pathways in the cells. We have not yet elucidated the molecular mechanism of this attenuated response to leptin. However, we do know that leptin receptor levels were not changed under these circumstances (data not shown), suggesting that some aspect of intracellular signaling pathways had been disturbed. For instances, SOCS3 elevation were observed in AMH11-55 cells pretreated with palmitate showing attenuated c-Fos responses to leptin. The elevated SOCS3 should be responsible for the disturbed response of the cells for leptin some extent, as previously reported [25, 26, 44, 45]. The palmitate induced SOCS3 elevation seemed to be mediated by TLR4. This results were consistent with the previous report that LPS stimulates SOCS3 expression in mouse brain and N-1 hypothalamic neurons via TLR4 [46]. On the other hand, we did not observe significant changes in the mRNA levels of PTP1B (Ptprn1), TCPTP (Ptpn2) and LAR (Ptprf), which cause leptin or insulin resistances [29, 31, 47, 48], in the current experimental condition. Further study will be necessary to elucidate whether other unknown factors are involved in the cellular leptin resistance and to determine to what extent this attenuation of *in vitro* responsiveness to leptin mirrors the leptin-resistant state *in vivo*.

Finally, in this study we selected the clones based on their leptin responsiveness: clones that more completely preserved leptin signaling were selected, because these cells were considered to be more suitable for evaluating leptin resistance. By selecting the clones by other criteria, it would be possible to obtain clones suitable for other purposes; however, this is beyond the scope of this study.

In summary, we developed adult mouse hypothalamus—derived cell lines (AMH cells). The cell lines we established exhibited moderate responses to leptin, insulin, NPY, adrenaline, and muscarine. These cell lines should be a useful tool for dissecting the molecular events induced by these appetite-controlling substances, and for finding a way to resolve the disturbance of signaling in the leptin-resistant state, although they have drawbacks including de-differentiated nature and lack of long-time stability.

## Supporting Information

S1 Fig STAT3 phosphorylation induced by leptin treatment of AMH cells. a. Stat3 phosphorylation was induced by addition of 100 nM leptin for 15 min in several clones of AMH cells. The images were arranged in numerical order. b-d. Stat3 (b), Erk1, 2 (c), and Akt (d) phosphorylation was induced by addition of 100 nM leptin for 15 min in clone 27, 55, 57, 59 of the hypothalamus-derived cell lines.
(PDF)

S2 Fig Intracellular Ca^2+^ concentrations following addition of known peptide hormones. Intracellular Ca^2+^ levels evoked by NPY (a), AVP (b), TRH (c), PYY3-36 (d), CCK-33 (e), NMS (f), orexin B (g), NMU (h), PP (i), neurotensin (j), ghrelin (k), LHRH (l), and kisspeptin (m) in AMH11-55 cells.
(PDF)

S3 Fig Intracellular Ca^2+^ concentration following addition of known neurotransmitters. Intracellular Ca^2+^ levels evoked by adrenaline (a), muscarine (b), histamine (c), glutamate (d), serotonin (e), melatonin (f), methoxamine (g), clonidine (h) and muscarine (i) in AMH11-55 cells.
(PDF)

S4 Fig Intracellular cAMP concentration following addition of known neurotransmitters or peptide hormones in clone 11–57. a, b. Intracellular cAMP concentration following addition of known neurotransmitters (a) and peptide hormones (c). n = 4, **: *p* < 0.01, **: *p* < 0.05 relative to forskolin. b, d. Intracellular cAMP concentration following addition of known neurotransmitters (b) and peptide hormones (d) with 5 uM forskolin. n = 4, **: *p* < 0.01, **: *p* < 0.05 relative to forskolin.
(PDF)

S5 Fig c-Fos mRNA responses to leptin. c-Fos mRNA responses of clone 11–27 (a), 57 (b), and 59 (c) to the addition of 100 nM leptin to the incubation medium. *: *p* < 0.05, **: *p* < 0.01 relative to 0 min. n = 4.
(PDF)

S6 Fig NPY and POMC mRNA responses to leptin. NPY (a, c, e) and POMC (b, d, f) mRNA responses of clone 11–55 (a, b), 57 (c, d), and 59 (e, f) to the addition of 100 nM leptin to the incubation medium (DMEM with 10% FBS) at 2, 4, 6, and 8 hours. *: *p* < 0.05, **: *p* < 0.01 relative to vehicle. n = 6.
(PDF)

S7 Fig Sequencing analysis of RT-PCR-product of TLR4. RT-PCR products of TLR4 in 11–55 were sequenced. The sequences in red frames were matched with expected TLR4 sequence.
(PDF)

S1 Table Primers and Taqman probes. (PDF)

S2 Table The mRNA expression levels of neural peptide, receptors and neural markers in the colony of hypothalamus-derived cells. The values are presented as % expression to that of the hypothalamus. Agrp: Agouti-related peptide, Npy: Neuropeptide Y, Pomc: Proopiomelanocortin, Cart: Cocain and amphetamine regulated transcript, Ghrl: Ghrelin, Gnrh: Gonadotropin releasing hormone, Ghrh: Growth hormone, releasing hormone, Oxt: Oxytocin, Sst: Somatostatin, Lepr: Leptin receptor, Ghsr: Growth hormone secretagogue receptor, Nefl: Neurofilament L, Chga: Chromogranin A, Nse: Neuron-specific enolase, Cdh2: Neural cadherin, Syp: Synaptophyisin.
(PDF)

S3 Table The mRNA expression levels of neural peptide, receptors, and neural markers in the hypothalamus-derived cell line clones derived from colony 11. The values are presented as expression ratio to that of the hypothalamus (%).
(PDF)
