# Supplementary figures and images for: Establishment of Leptin-Responsive Cell Lines from Adult Mouse Hypothalamus
Source: PLoS One. 2016 Feb 5;11(2):e0148639. doi: 10.1371/journal.pone.0148639 (PMC4744015; doi:10.1371/journal.pone.0148639)

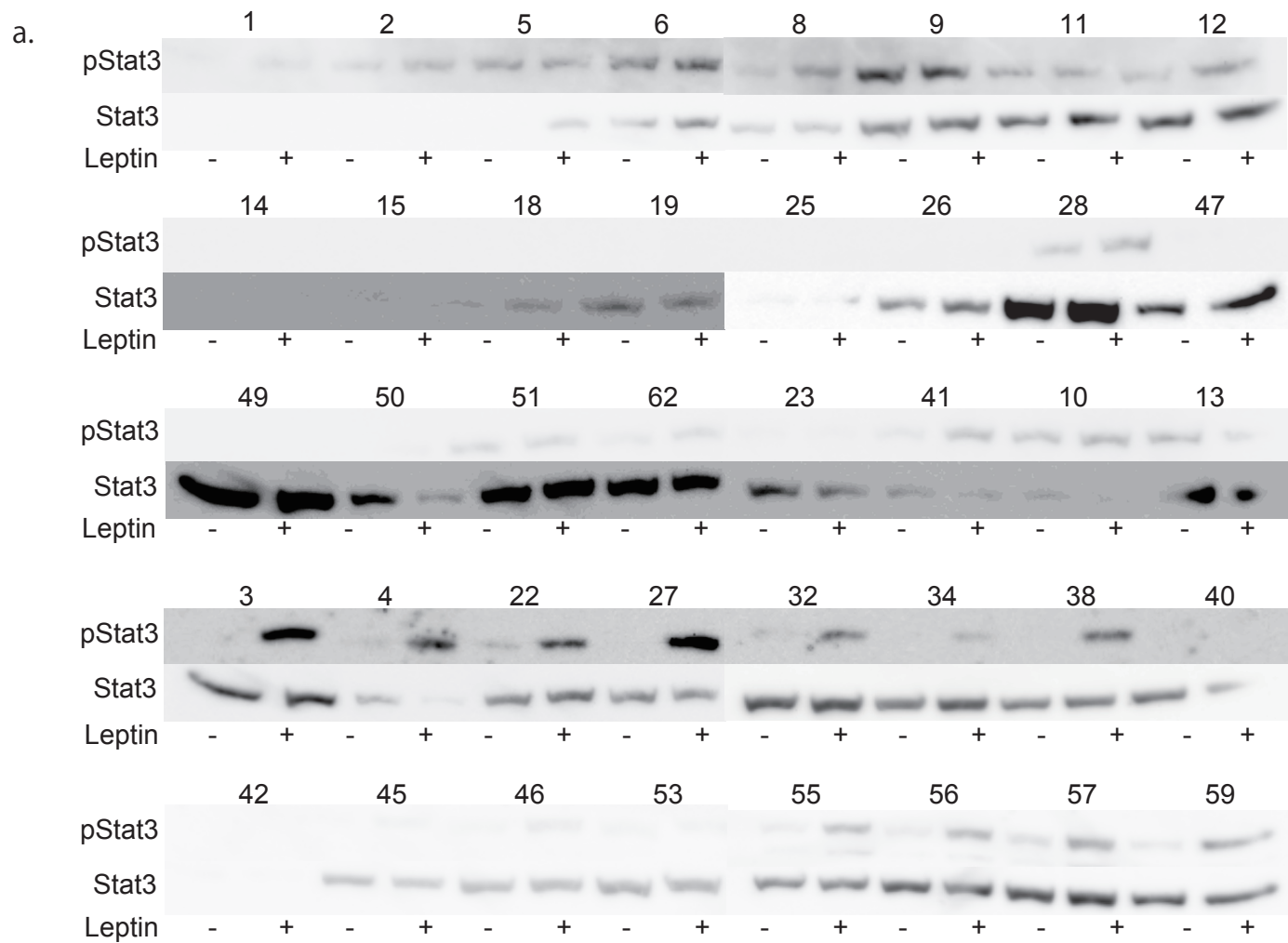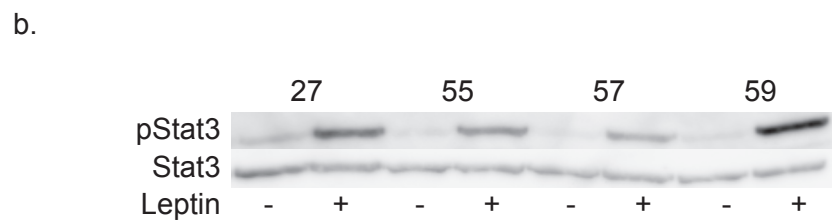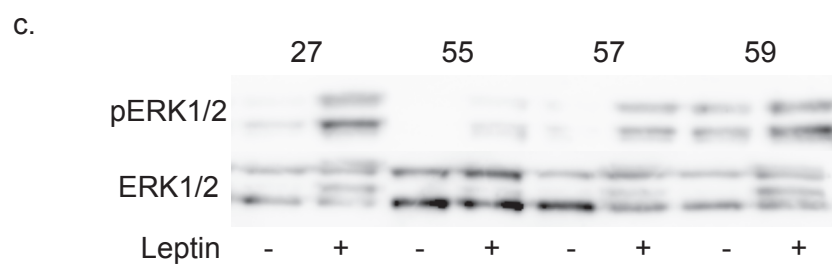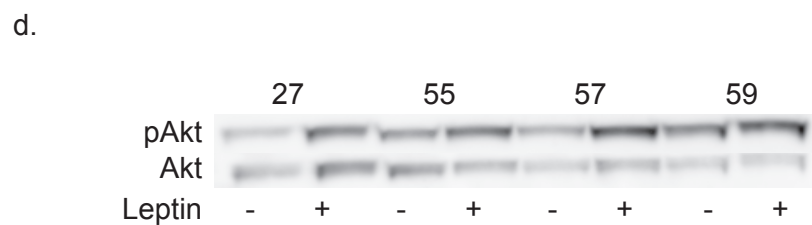

S1 Fig.

Supplement: S1 Fig — a. Stat3 phosphorylation was induced by addition of 100 nM leptin for 15 min in several clones of AMH cells. The images were arranged in numerical order. b-d. Stat3 (b), Erk1, 2 (c), and Akt (d) phosphorylation was induced by addition of 100 nM leptin for 15 min in clone 27, 55, 57, 59 of the hypothalamus-derived cell lines. (PDF) [file pone.0148639.s001.pdf]

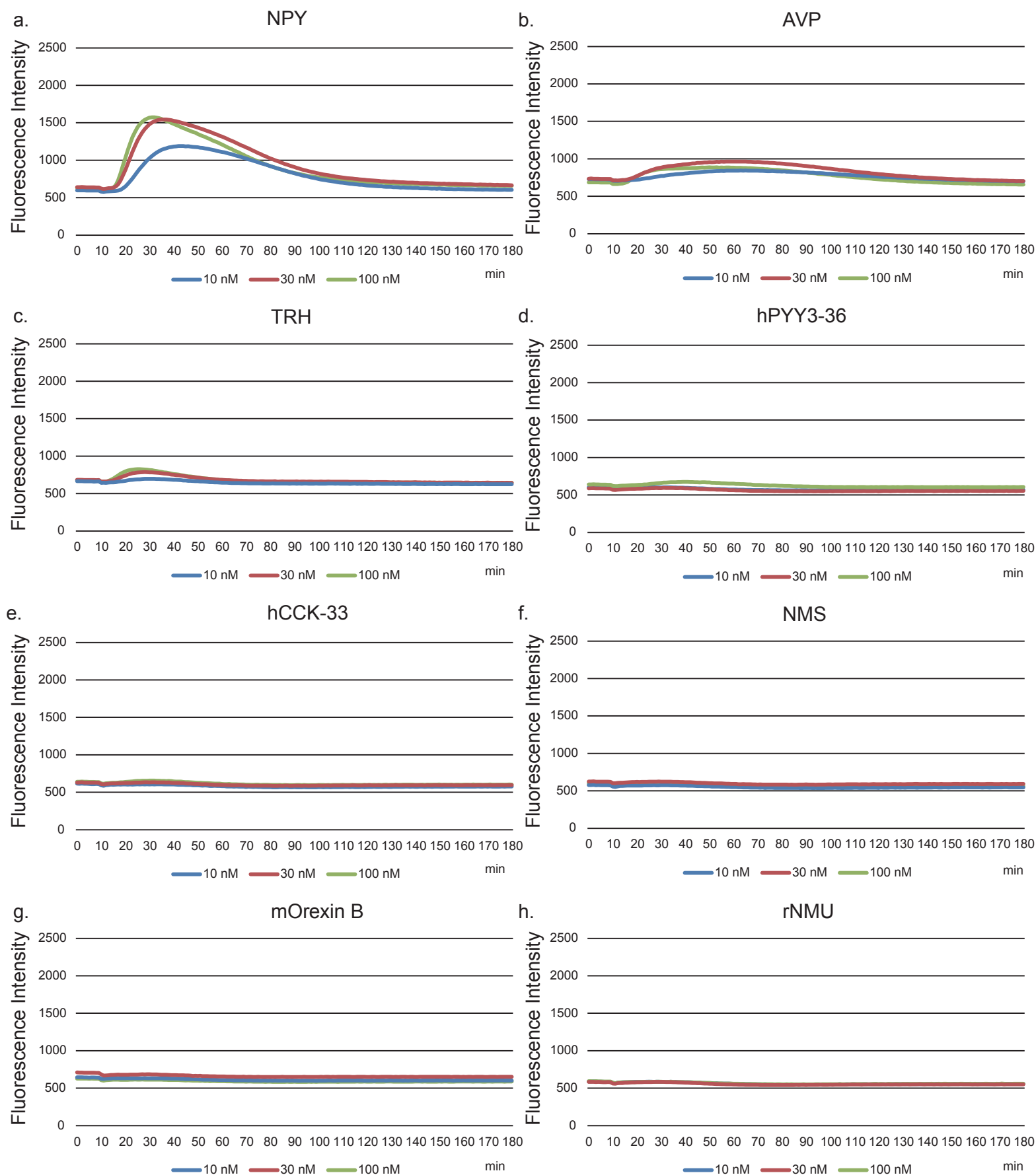

S2 Fig.

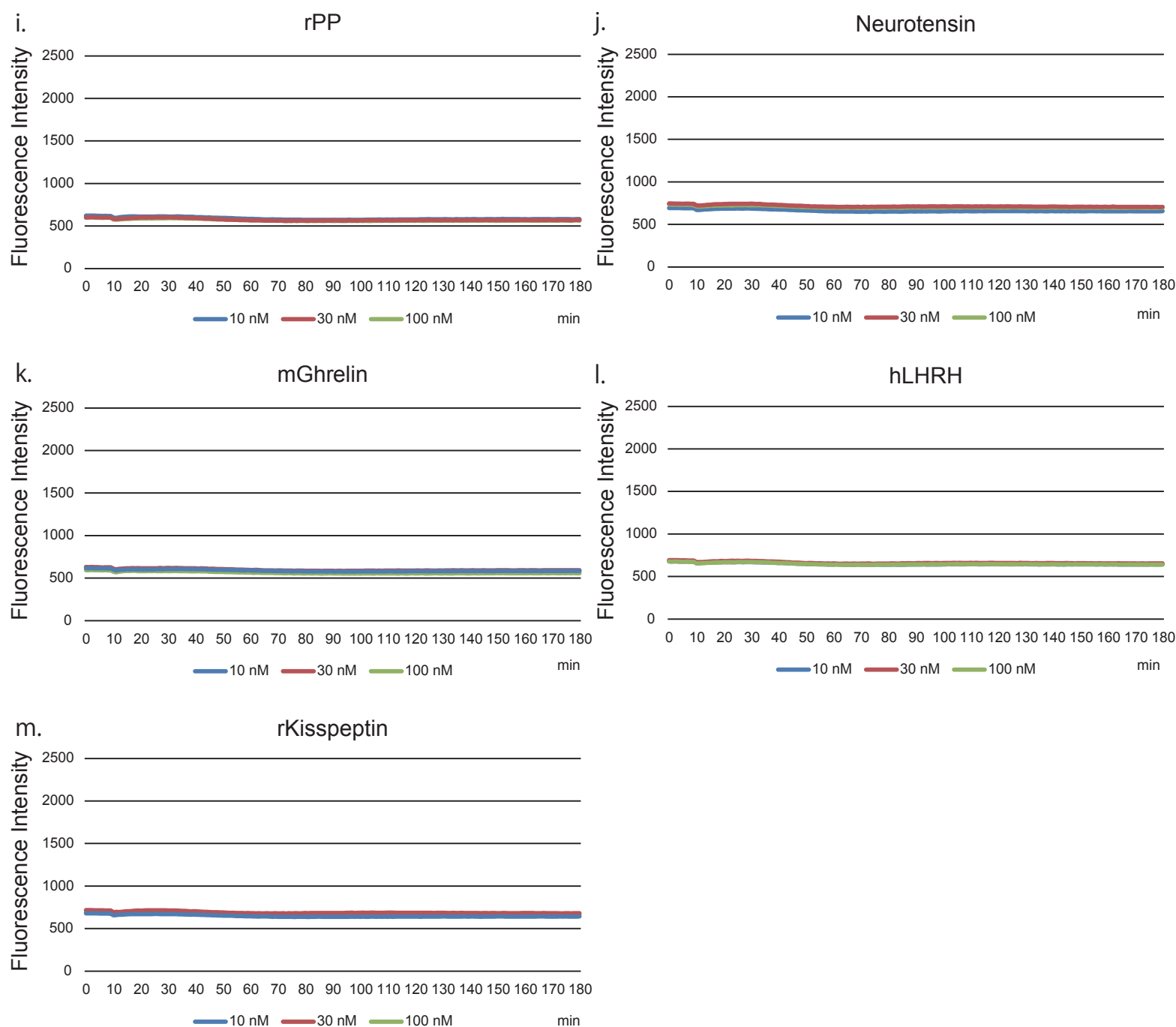

S2 Fig.

Supplement: S2 Fig — Intracellular Ca2+ levels evoked by NPY (a), AVP (b), TRH (c), PYY3-36 (d), CCK-33 (e), NMS (f), orexin B (g), NMU (h), PP (i), neurotensin (j), ghrelin (k), LHRH (l), and kisspeptin (m) in AMH11-55 cells. (PDF) [file pone.0148639.s002.pdf]

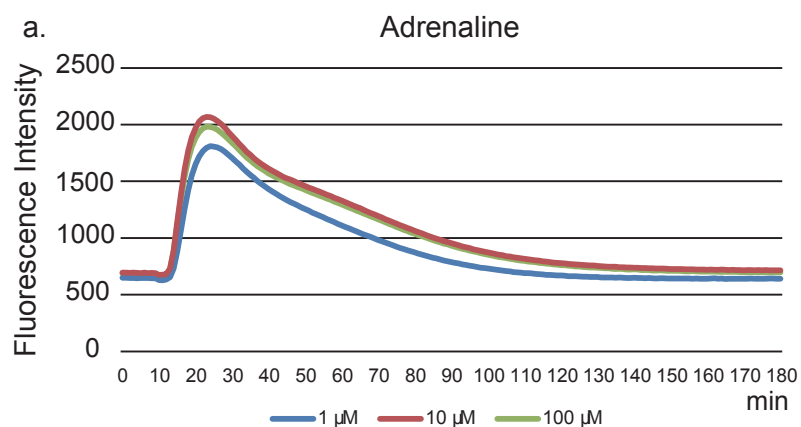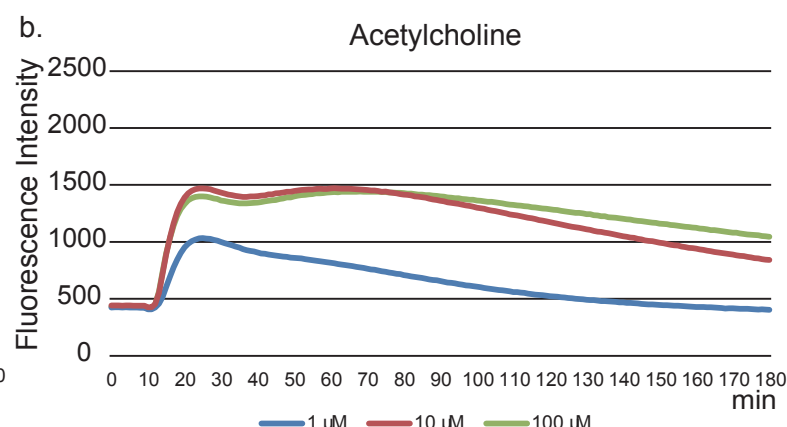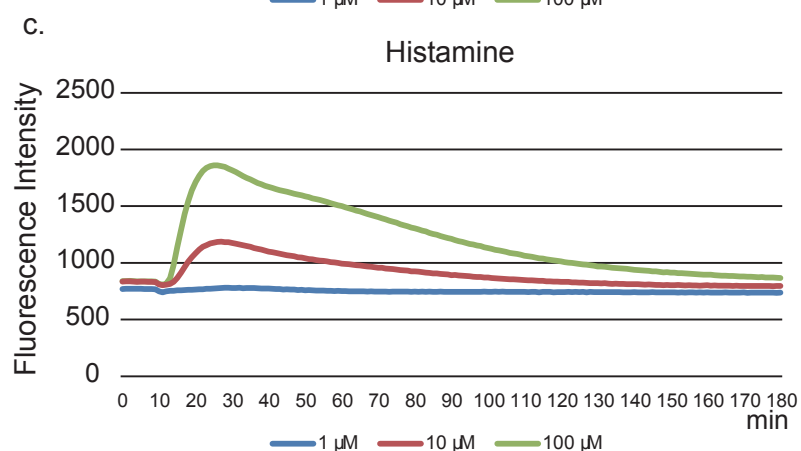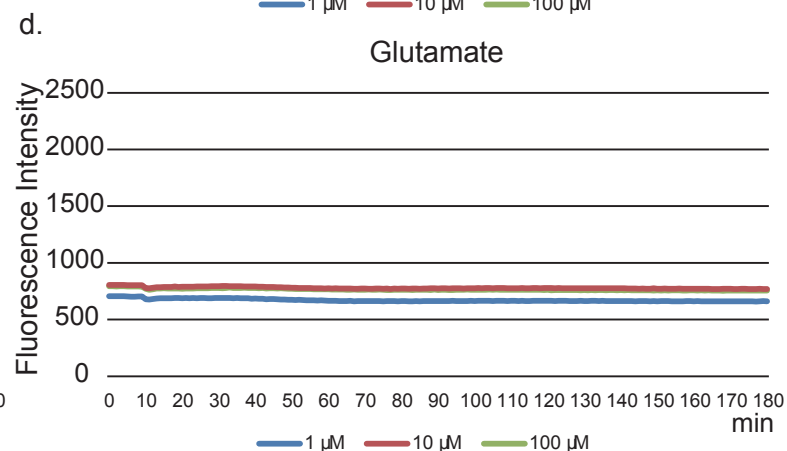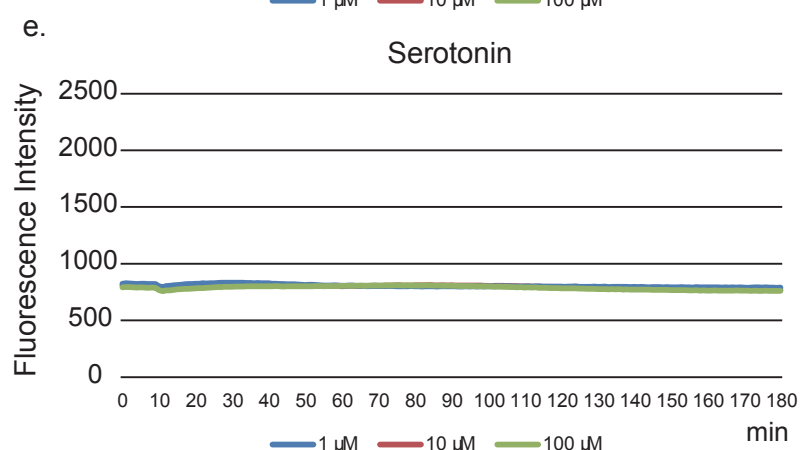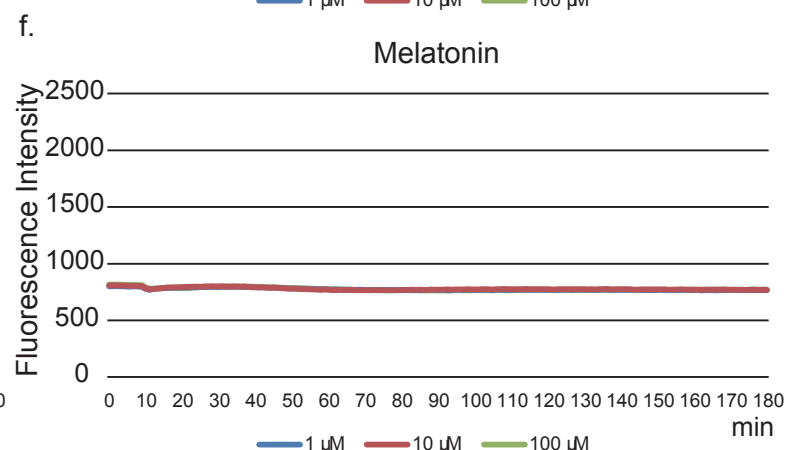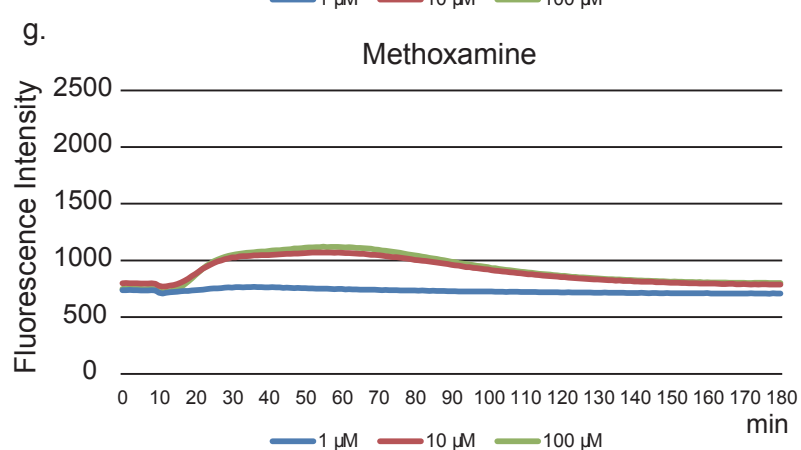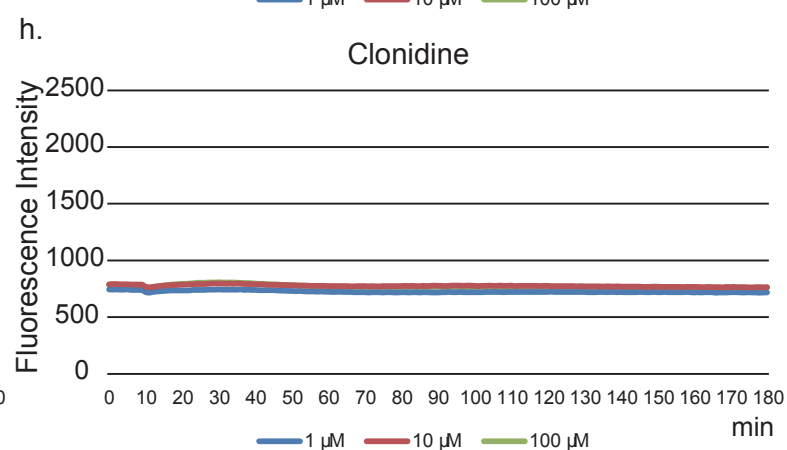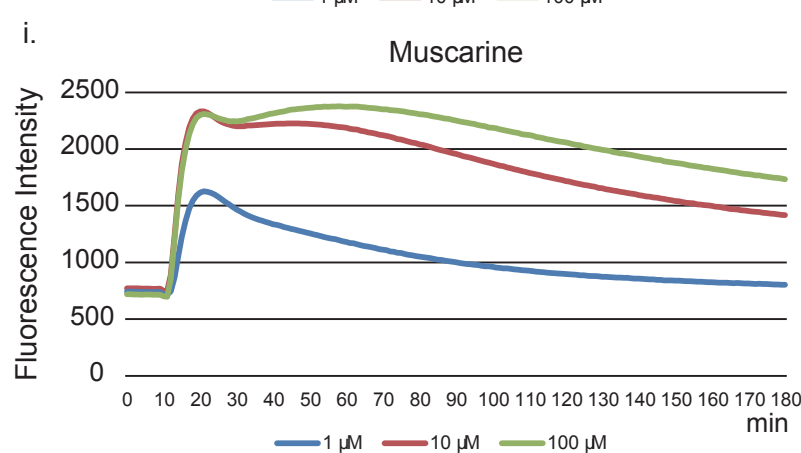

Supplement: S3 Fig — Intracellular Ca2+ levels evoked by adrenaline (a), muscarine (b), histamine (c), glutamate (d), serotonin (e), melatonin (f), methoxamine (g), clonidine (h) and muscarine (i) in AMH11-55 cells. (PDF) [file pone.0148639.s003.pdf]

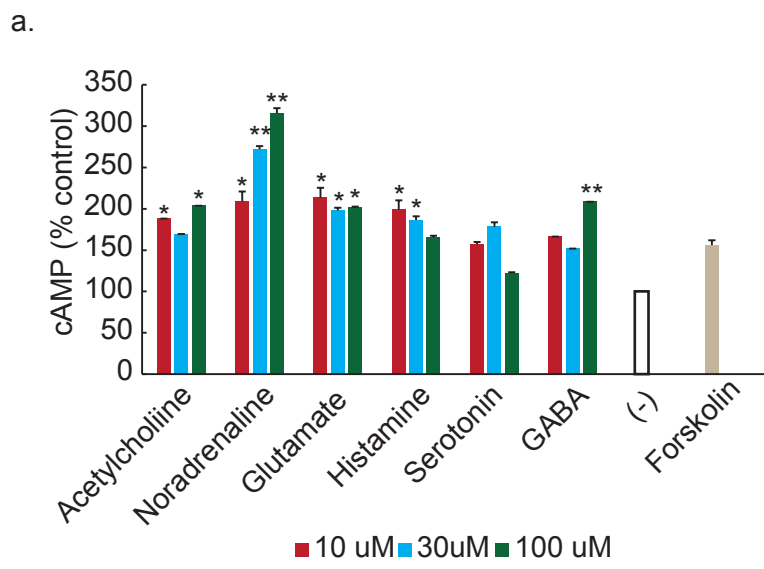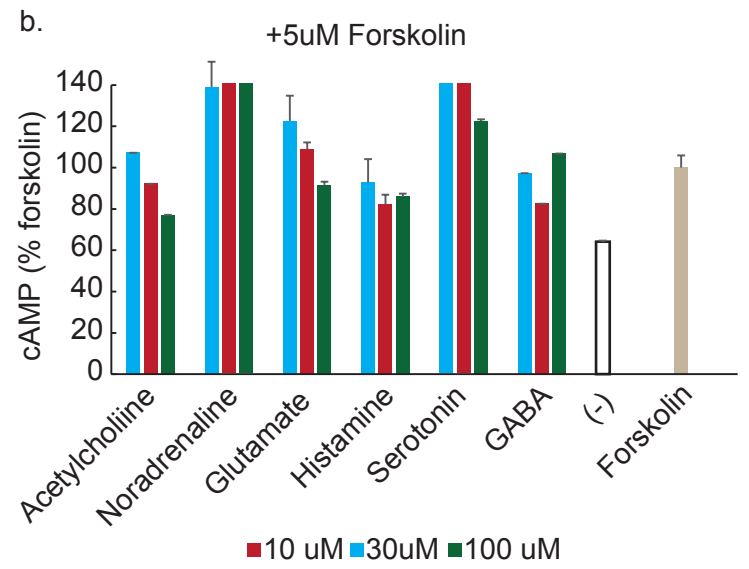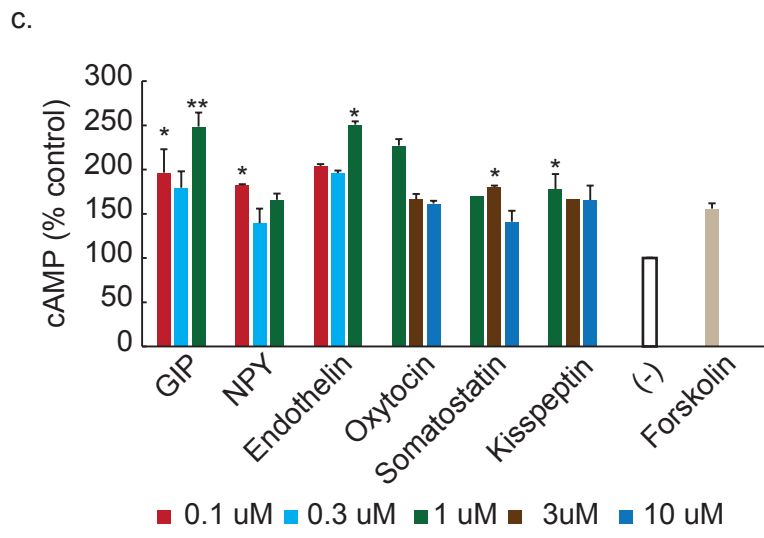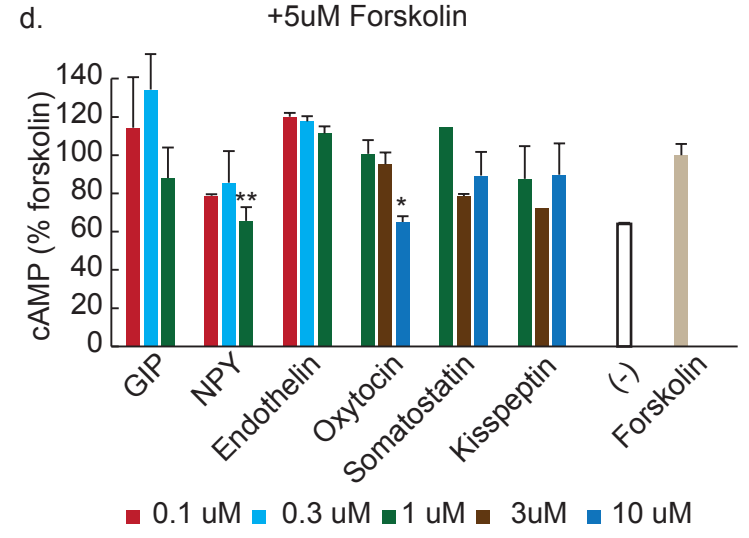

Supplement: S4 Fig — a, b. Intracellular cAMP concentration following addition of known neurotransmitters (a) and peptide hormones (c). n = 4, **: p < 0.01, **: p < 0.05 relative to forskolin. b, d. Intracellular cAMP concentration following addition of known neurotransmitters (b) and peptide hormones (d) with 5 uM forskolin. n = 4, **: p < 0.01, **: p < 0.05 relative to forskolin. (PDF) [file pone.0148639.s004.pdf]

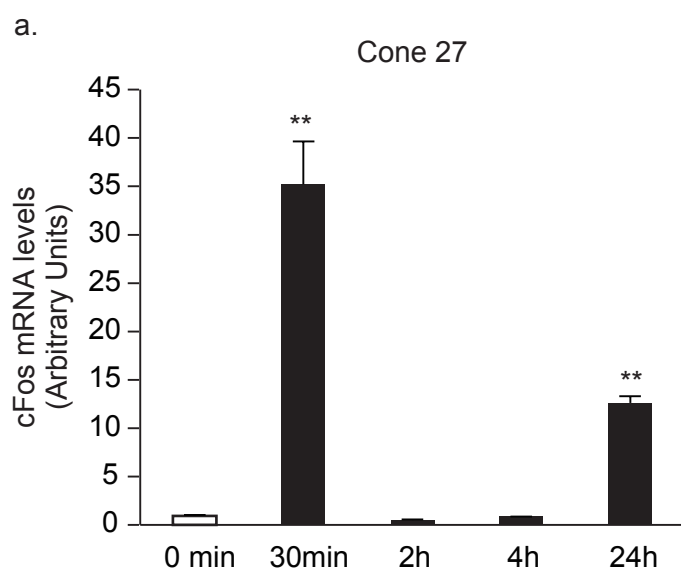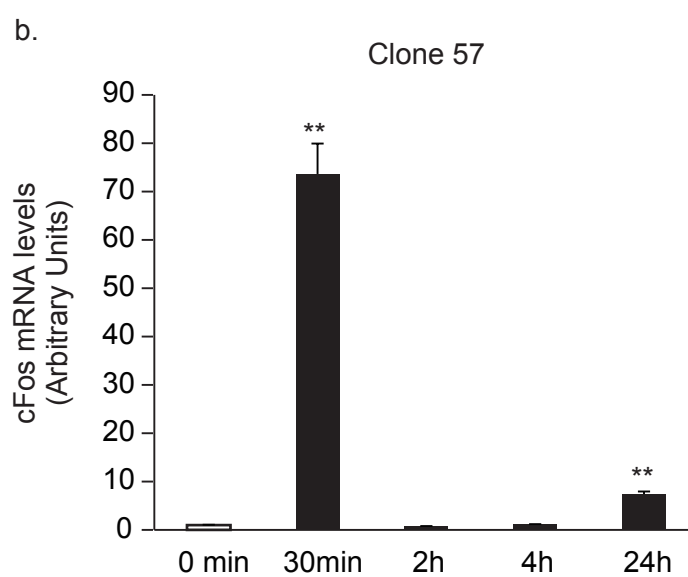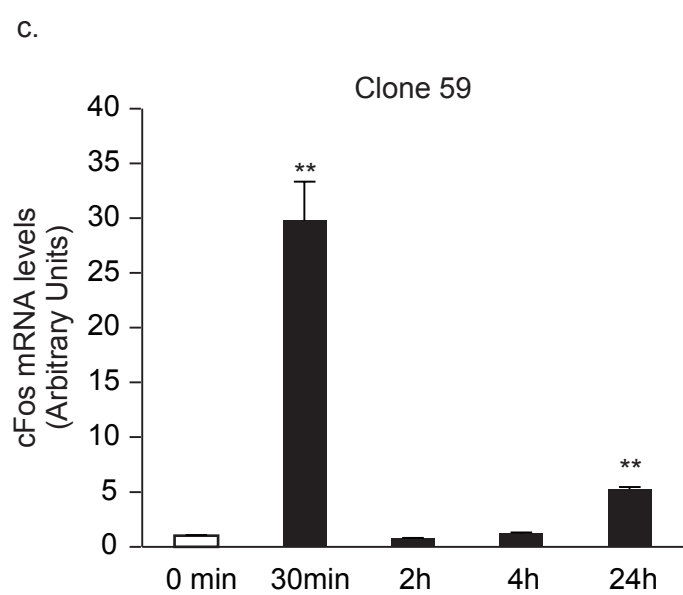

Supplement: S5 Fig — c-Fos mRNA responses of clone 11–27 (a), 57 (b), and 59 (c) to the addition of 100 nM leptin to the incubation medium. *: p < 0.05, **: p < 0.01 relative to 0 min. n = 4. (PDF) [file pone.0148639.s005.pdf]

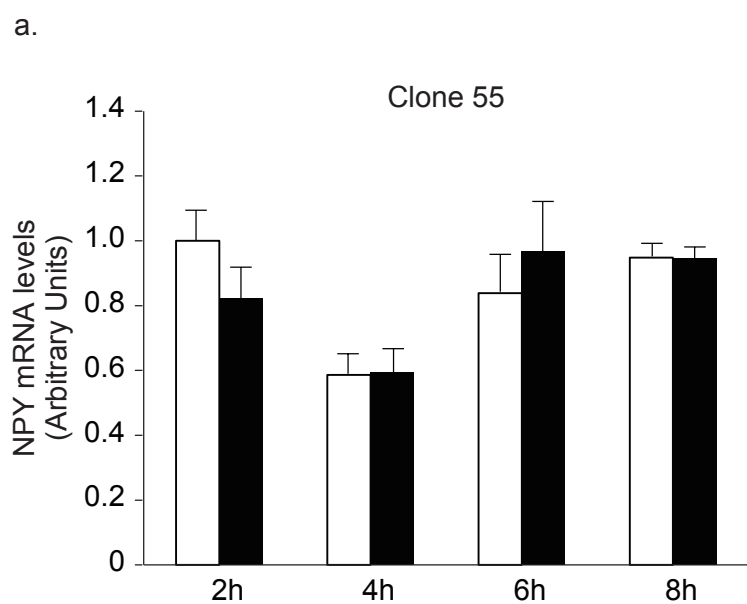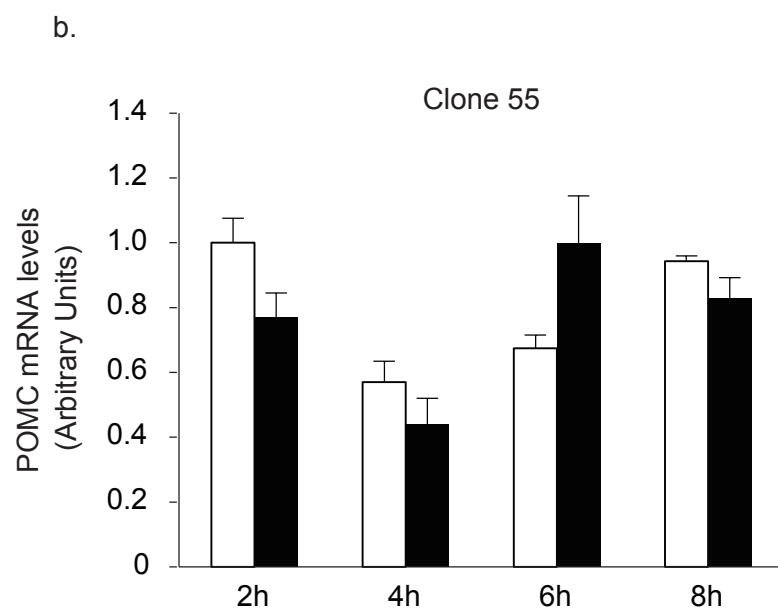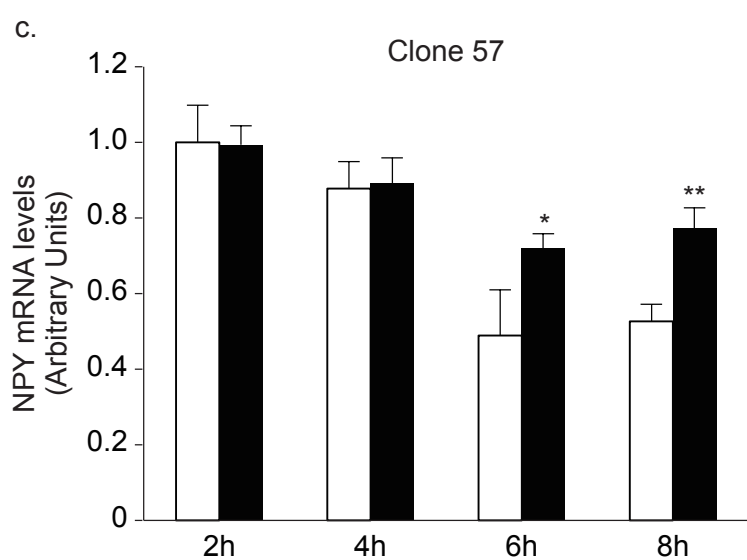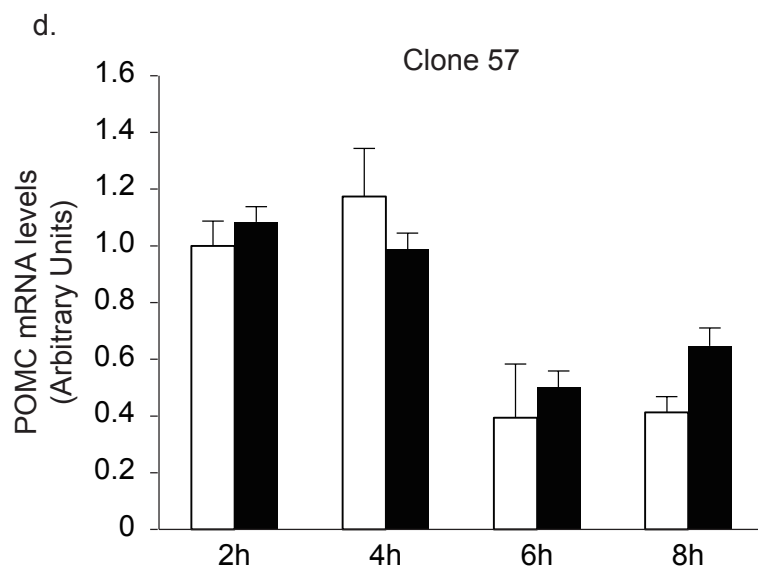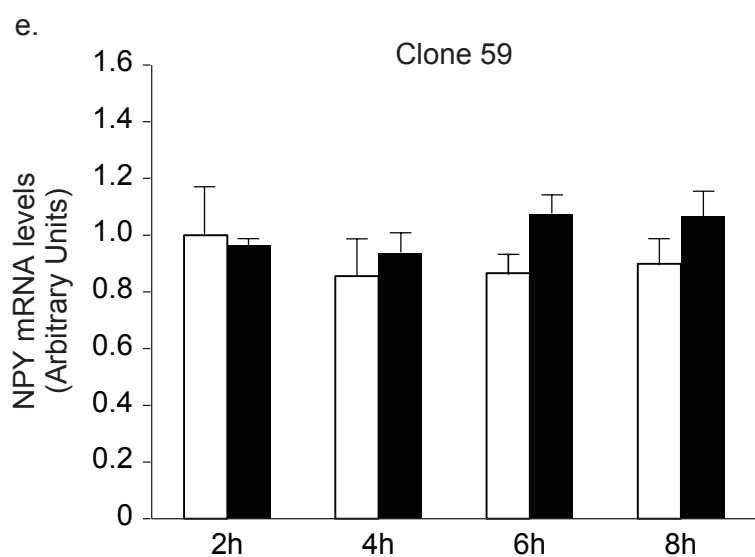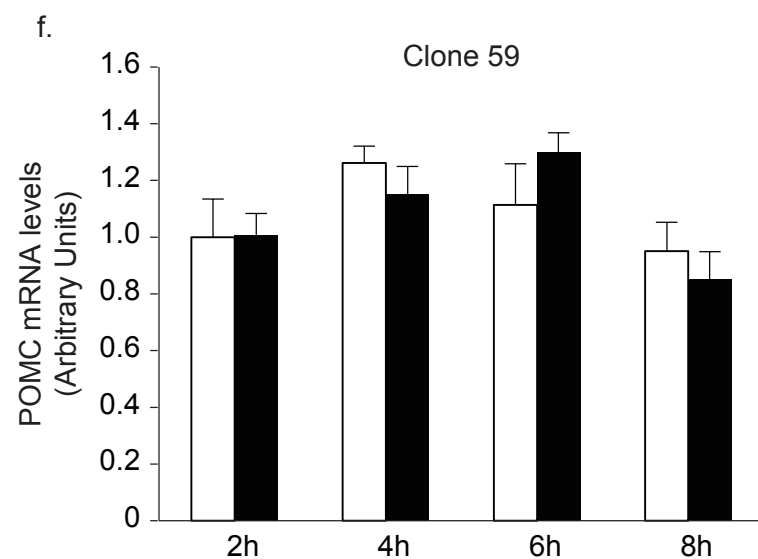

S6 Fig.

Supplement: S6 Fig — NPY (a, c, e) and POMC (b, d, f) mRNA responses of clone 11–55 (a, b), 57 (c, d), and 59 (e, f) to the addition of 100 nM leptin to the incubation medium (DMEM with 10% FBS) at 2, 4, 6, and 8 hours. *: p < 0.05, **: p < 0.01 relative to vehicle. n = 6. (PDF) [file pone.0148639.s006.pdf]

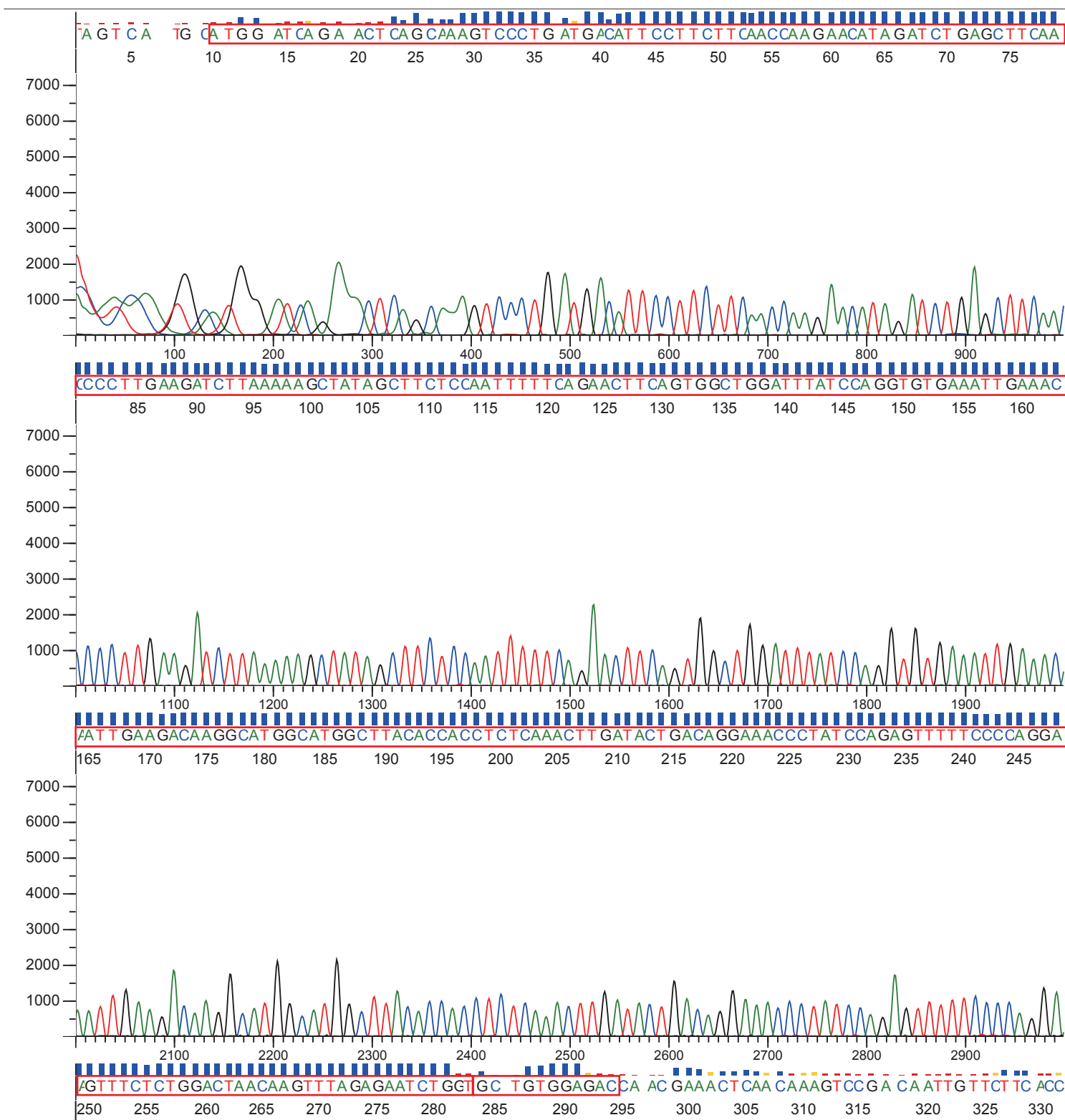

S7 Fig.

Supplement: S7 Fig — RT-PCR products of TLR4 in 11–55 were sequenced. The sequences in red frames were matched with expected TLR4 sequence. (PDF) [file pone.0148639.s007.pdf]
