# Supplementary material for: Establishment of Leptin-Responsive Cell Lines from Adult Mouse Hypothalamus
Source: PLoS One. 2016 Feb 5;11(2):e0148639. doi: 10.1371/journal.pone.0148639 (PMC4744015; doi:10.1371/journal.pone.0148639)
Supplement: S1 Table — (PDF) [file pone.0148639.s008.pdf]

AgRP	sense	5'-GGCACAAGAGACCAGGACATC-3'
	antisense	5'-GAACACAACTCAGCAACATTGCA-3'
	Taqman probe	5'-CAAAGATCAGCAAGCAAAGGCCATGC-3'
NPY	sense	5'-TGGACTGACCCTCGCTCTAT-3'
	antisense	5'-CAACAACAACAAGGGAAATGG-3'
POMC	sense	5'-GGGTCCCTCCAATCTTGTTT-3'
	antisense	5'-TTTTCAGTCAGGGGCTGTTC-3'
CART	sense	5'-GCCCTGGACATCTACTCTGC-3'
	antisense	5'-TGAAGCAACAGGGAAAGAGC-3'
ghrelin	sense	5'-GCATGCTCTGGATGGACATG-3'
	antisense	5'-TGGTGGCTTCTTGGATTCCT-3'
	Taqman probe	5'-AGCCCAGAGCACCAGAAAGCCCA-3'
Gnrh	sense	5'-AGCACTGGTCCTATGGGTTG-3'
	antisense	5'-CTTCTGCCTGGCTTCCTCTT-3'
Ghrh	sense	5'-AGGATGCAGCGACACGTAGA-3'
	antisense	5'-TCTCCCCTTGCTTGTTCATGA-3'
	Taqman probe	5'-CCACCAACTACAGGAAACTCCTGAGCCA-3'
Oxt	sense	5'-TGCTTGGCTTACTGGCTCTGA-3'
	antisense	5'-GGAGACACTTGCGCATATCCA-3'
	Taqman probe	5'-TCGGCCTGCTACATCCAGAACTGCC-3'
Sst	sense	5'-CCACCGGGAAACAGGAACT-3'
	antisense	5'-GGGCATCATTCTCTGTCTGGTT-3'
	Taqman probe	5'-CAAGTACTTCTTGGCAGAGC-3'
Lepr	sense	5'-TTGGAAGCCCCTGACGAAAAA-3'
	antisense	5'-TGAAATGGGTTCAGGCTCCAG-3'
Ghsr	sense	5'-AGGGACCAGAACCACAAACAGA-3'
	antisense	5'-CAGCAGAGGATGAAAGCAAACA-3'
	Taqman probe	5'-AAGATGCTTGCTGTGGTGG-3'
Nefl	sense	5'-GACAAGCAGAATGCAGACATTAGC-3'
	antisense	5'-CCTGGCCATCTCGCTCTTC-3'
	Taqman probe	5'-CCATGCAGGACACAATCAACAAACTGGA-3'
Chga	sense	5'-GACACCAAGGTGATGAAGTGC-3'
	antisense	5'-GCATCAGGGCTCTGGTTCTC-3'
Nse	sense	5'-ATCTGAACGTCTGGCGAAGT-3'
	antisense	5'-TCACAGCACACTGGGATTTC-3'
Cdh2	sense	5'-AGCCCGGTTTCACTTGAGAG-3'
	antisense	5'-TCCGTGACAGTTAGGTTGGC-3'
Syp	sense	5'-GCAGTGGGTCTTTGCCATCT-3'
	antisense	5'-CCGAGGAGGAGTAGTCACCA-3'
Tag	sense	5'-AAACACTGCAGGCCAGATTT-3'
	antisense	5'-AAATGAGCCTTGGGACTGTG-3'
cfos	sense	5'-GGGCAAAGTAGAGCAGCTATCTC-3'
	antisense	5'-CTGTCTCCGCTTGGAGTGTATC-3'
	Taqman probe	5'-CCTCCTCCGATTCCGGCACTTGGCT-3'
Socs3	sense	5'-AGCCCCAAGGCCGGAGAT-3'
	antisense	5'-GGGAAACTTGCTGTGGGTGAC-3'
	Taqman probe	5'-CCTGCATTTCCACTACACCACATGGCCT-3'
Ptpn1	sense	5'-GTTGGAGTTGGAAAACCTGACTAC-3'
	antisense	5'-GAGCCGTCGTAGCCTTTCTATC-3'
	Taqman probe	5'-CCTGCATTTCCACTACACCACATGGCCT-3'
Ptpn2	sense	5'-CTTCCTTCTAAGGTGCAGGATACT-3'
	antisense	5'-GAGCCGTCGTAGCCTTTCTATC-3'
	Taqman probe	5'-CCGTAGAATGCTCTCACTGCTCTCCTCC-3'
Ptprf	sense	5'-GGCTGAAGGACTCCCTGTTG-3'
	antisense	5'-GGGTGGTCTCGCATACCTGG-3'
	Taqman probe	5'-AAGCCTTCGCATCTCCACAGGGTCAGA-3'
TLR4	sense	5'-AATCCCTGCATAGAGGTAGTTCC-3'
	antisense	5'-GTCTCCACAGCCACCAGATT-3'
