# Supplementary material for: Establishment of Leptin-Responsive Cell Lines from Adult Mouse Hypothalamus
Source: PLoS One. 2016 Feb 5;11(2):e0148639. doi: 10.1371/journal.pone.0148639 (PMC4744015; doi:10.1371/journal.pone.0148639)
Supplement: S2 Table — The values are presented as % expression to that of the hypothalamus. Agrp: Agouti-related peptide, Npy: Neuropeptide Y, Pomc: Proopiomelanocortin, Cart: Cocain and amphetamine regulated transcript, Ghrl: Ghrelin, Gnrh: Gonadotropin releasing hormone, Ghrh: Growth hormone, releasing hormone, Oxt: Oxytocin, Sst: Somatostatin, Lepr: Leptin receptor, Ghsr: Growth hormone secretagogue receptor, Nefl: Neurofilament L, Chga: Chromogranin A, Nse: Neuron-specific enolase, Cdh2: Neural cadherin, Syp: Synaptophyisin. (PDF) [file pone.0148639.s009.pdf]

	Agrp	Npy	Pomc	Cart	Ghrl	Gnrh	Ghrh	Oxt	Sst	Lepr	Ghsr	Nefl	Chga	Nse	Cdh2	Syp
colony 1	1084.7	26.4	92.3	2058.4	41.1	67.3	167.5	184.3	9.32	114.0	0.057	15.0	0.8	13.7	238.1	0.09
colony 2	1350.9	35.7	215.1	3130.7	83.3	115.0	717.4	651.7	0.93	150.8	ND	3.1	1.1	9.8	902.2	0.22
colony 3	934.3	5.5	75.1	1336.8	54.2	67.9	103.5	184.6	ND	121.5	ND	0.2	0.6	8.6	262.7	0.09
colony 6	1126.5	21.4	119.5	2524.9	43.3	105.1	372.6	392.4	1.49	149.3	ND	6.9	1.4	10.0	286.2	0.17
colony 8	1693.4	7.2	68.5	1659.2	60.8	67.4	98.5	262.6	0.34	105.7	ND	5.2	0.4	11.3	268.8	0.06
colony 9	1282.4	27.4	178.3	3003.3	74.4	90.1	288.8	132.0	0.78	135.0	0.264	4.2	0.7	15.5	604.9	0.12
colony 10	1438.0	14.2	71.5	1337.2	51.6	53.1	121.3	220.8	0.54	125.9	ND	1.1	0.5	13.7	749.6	0.08
colony 11	1999.1	31.9	332.7	4217.7	230.5	146.3	1306.8	1630.9	1.78	234.1	0.076	7.4	4.6	16.2	134.6	0.48
colony 16	1197.8	6.4	43.8	1691.7	47.3	34.3	119.2	135.1	1.51	162.0	0.270	4.7	2.0	8.5	171.2	0.16
colony 17	1024.5	20.3	196.9	2615.5	55.9	127.5	514.6	570.7	0.76	177.8	ND	0.1	1.4	4.3	387.4	0.23
colony 18	1184.5	64.7	207.6	1185.9	32.6	37.3	110.5	99.6	0.81	175.3	ND	3.5	0.4	4.0	131.1	0.07
colony 19	1475.2	15.6	119.6	2740.7	42.5	124.2	162.1	168.6	ND	215.9	0.152	21.3	2.2	16.0	158.7	0.22
colony 20	1106.3	7.7	76.6	2666.4	28.4	34.8	ND	99.7	ND	195.9	0.055	0.3	9.9	8.0	198.1	1.85
colony 21	1536.2	14.8	130.6	2501.8	119.4	107.4	207.9	168.7	0.78	127.5	ND	0.5	0.6	14.1	228.3	0.06
colony 22	1197.3	5.4	41.3	2206.8	51.4	48.0	69.4	120.0	8.73	157.3	ND	0.4	0.8	12.1	243.8	0.10
colony 23	1076.7	9.3	87.0	1957.1	67.3	53.5	111.9	177.6	1.61	117.7	ND	ND	0.4	11.7	230.2	0.07
colony 24	1395.7	22.0	111.5	3091.2	72.5	100.5	540.0	320.3	2.39	147.6	ND	2.2	1.0	9.0	407.4	0.19
colony 25	579.3	15.9	72.3	1595.3	22.6	56.4	120.7	136.5	1.54	131.0	ND	1.9	1.8	7.0	154.7	0.30
colony 26	1130.5	5.9	56.5	2055.8	59.8	65.9	106.2	151.6	1.27	165.1	ND	6.8	3.3	11.6	261.5	0.34
colony 27	978.9	6.2	60.8	1905.4	76.4	52.9	95.7	277.8	0.45	131.6	0.431	3.3	33.3	9.2	276.6	8.25
colony 28	1650.4	57.4	65.6	1053.1	56.6	29.0	23.6	ND	0.40	105.0	0.044	0.2	15.3	6.5	225.2	2.31
colony 29	1764.6	23.0	170.1	2694.6	70.2	110.1	292.9	209.0	9.01	138.6	0.188	2.4	0.7	8.1	210.6	0.11
colony 30	1487.1	21.6	113.1	1731.8	46.4	67.0	179.7	183.4	0.49	118.9	ND	3.7	0.6	8.9	206.1	0.09
colony 31	1592.1	12.6	96.2	1888.6	73.0	95.3	140.1	118.0	1.89	144.5	ND	7.1	0.8	15.6	178.4	0.08
colony 34	761.8	67.8	299.9	1243.5	49.5	58.8	402.8	623.2	ND	193.2	ND	0.2	0.7	2.1	318.3	0.14
colony 35	2023.8	37.1	54.6	679.9	53.4	17.0	15.9	131.6	0.46	120.6	ND	0.3	0.5	11.9	553.6	0.07
colony 37	330.1	2.8	98.8	3186.0	47.6	38.9	22.5	169.0	0.78	303.7	0.047	0.4	4.7	4.4	200.9	0.71
colony 38	1743.8	58.4	98.1	1070.5	76.5	24.8	49.5	ND	1.20	160.5	ND	0.1	0.6	5.7	290.9	0.05
colony 39	724.7	11.0	415.8	2261.2	199.3	109.4	143.9	280.8	ND	233.2	1.386	16.2	57.7	4.3	662.2	17.7
colony 40	314.8	9.0	181.2	1134.4	114.9	100.0	594.2	803.3	ND	125.7	0.147	14.3	101.7	5.8	335.0	12.9
