# Supplementary material for: Establishment of Leptin-Responsive Cell Lines from Adult Mouse Hypothalamus
Source: PLoS One. 2016 Feb 5;11(2):e0148639. doi: 10.1371/journal.pone.0148639 (PMC4744015; doi:10.1371/journal.pone.0148639)
Supplement: S3 Table — The values are presented as expression ratio to that of the hypothalamus (%). (PDF) [file pone.0148639.s010.pdf]

	Agrp	Npy	Pomc	Cart	Ghrl	GnRH	Ghrh	Avp	Oxt	Sst	Lepr	Ghsr	Nefl	Chga	Nse	Cdh2	Syp
11−1	58.4	7.7	5.9	35.7	81	88.8	2.5	ND	0.7	0.12	76.6	ND	0.06	0.94	1.78	89	0.16
11−2	61.4	9.3	6.3	47.5	160	85.5	19.1	ND	1.3	0.08	113.0	ND	0.01	1.54	2.57	367	0.22
11−3	47.0	6.8	6.8	64.4	352	206.1	15.4	ND	1.3	ND	277.4	ND	0.05	2.47	4.67	234	0.33
11−4	19.7	7.4	16.4	49.5	118	93.5	5.0	ND	0.7	0.11	188.4	ND	0.04	0.47	0.72	163	0.18
11−5	24.4	13.4	8.5	60.6	194	163.5	64.5	ND	3.9	ND	127.0	0.05	0.04	2.20	1.71	148	0.26
11−6	30.2	4.6	2.3	28.8	104	63.7	3.7	0.002	1.3	0.10	104.5	ND	4.37	1.09	3.19	111	0.11
11−8	127.2	5.7	13.9	37.8	257	73.6	16.3	ND	0.5	ND	72.7	ND	0.02	0.30	7.86	293	0.94
11−9	23.3	7.4	17.5	34.4	140	98.0	5.4	ND	0.9	ND	187.2	ND	0.01	1.27	2.64	245	0.19
11−10	23.0	6.8	5.0	54.4	135	122.7	5.9	ND	0.6	ND	245.0	ND	0.02	2.18	1.60	404	0.23
11−11	85.7	13.6	25.8	52.4	361	163.3	56.5	0.003	0.9	0.12	71.7	ND	0.02	0.45	1.01	219	0.62
11−12	21.3	6.7	4.4	34.2	54	83.2	1.4	ND	0.3	0.08	124.7	ND	0.72	1.45	1.85	96	0.19
11−13	40.5	4.5	4.7	41.5	126	58.4	4.6	ND	1.2	ND	113.2	ND	0.01	1.02	2.49	249	0.10
11−14	27.6	9.8	7.1	61.2	143	130.2	20.7	ND	2.1	ND	186.2	ND	ND	2.00	1.11	320	0.25
11−15	19.7	6.6	18.8	45.6	117	46.8	6.3	ND	0.2	ND	200.6	ND	0.01	0.60	1.46	286	0.23
11−16	36.2	7.3	20.3	41.7	135	78.1	11.8	ND	0.4	ND	179.4	ND	0.44	1.26	3.56	244	0.25
11−18	19.1	5.2	9.7	25.6	65	35.1	2.7	ND	0.4	ND	104.6	ND	0.48	0.85	0.93	69	0.13
11−19	20.7	3.2	2.7	30.2	124	46.4	0.5	ND	0.6	ND	91.2	ND	0.51	0.81	2.20	86	0.11
11−20	122.7	5.7	21.4	45.2	289	107.5	70.4	0.003	1.1	ND	46.7	0.04	1.88	0.37	2.70	236	1.12
11−22	202.4	16.7	32.8	77.7	1008	136.9	174.3	0.008	1.7	ND	80.1	ND	0.04	0.41	2.73	201	1.12
11−23	22.6	10.6	7.1	45.9	85	95.8	16.5	ND	1.4	ND	117.1	0.12	5.80	1.55	1.13	341	0.21
11−24	21.3	11.6	25.0	40.7	93	49.4	13.2	0.002	1.1	ND	168.0	ND	3.27	1.27	1.34	210	0.22
11−25	11.3	4.0	17.6	41.4	119	37.9	7.7	ND	0.1	ND	169.7	ND	ND	0.84	1.25	220	0.15
11−26	14.0	8.3	5.9	48.1	92	85.5	9.0	ND	0.9	0.14	139.9	ND	0.07	1.71	5.28	366	0.25
11−27	84.7	5.9	14.4	36.0	237	87.8	5.5	0.002	0.4	ND	76.3	0.07	0.30	0.39	6.05	296	0.94
11−28	5.3	4.6	4.0	28.3	50	28.3	3.8	0.001	1.2	ND	149.2	ND	2.08	1.11	1.62	291	0.15
11−32	48.4	2.7	3.3	47.5	144	64.9	0.4	ND	0.5	ND	177.3	0.03	0.06	1.22	1.88	81	0.16
11−34	77.0	5.9	15.2	33.5	119	34.4	2.1	ND	0.6	ND	187.7	ND	0.03	0.25	1.43	271	0.16
11−37	67.7	3.6	10.7	26.1	138	72.5	4.1	0.006	0.3	0.21	105.9	ND	2.95	0.35	1.67	298	0.66
11−38	135.7	13.2	26.6	57.2	445	139.8	82.1	ND	1.7	ND	51.2	ND	ND	0.33	3.74	541	0.28
11−40	92.5	14.8	16.1	53.9	157	87.7	34.0	ND	0.5	ND	347.6	ND	0.05	0.58	5.04	278	1.10
11−41	89.8	7.5	19.0	42.3	326	97.1	15.6	0.003	0.3	ND	55.2	0.16	1.33	0.47	4.44	234	1.10
11−42	115.4	13.4	22.4	41.2	257	133.5	73.1	0.005	2.1	ND	44.1	0.03	ND	0.20	5.21	457	1.00
11−43	113.5	8.2	16.7	39.1	373	176.3	18.7	0.006	1.0	ND	50.0	0.02	0.02	0.29	2.78	268	0.96
11−45	158.8	13.7	26.4	53.7	345	107.7	18.5	0.010	1.0	ND	53.5	0.01	0.01	0.39	2.78	303	1.17
11−46	422.4	9.1	12.2	52.8	223	52.1	10.2	ND	0.3	ND	104.1	ND	0.02	0.22	5.05	181	0.98
11−47	137.1	17.2	31.5	67.3	378	109.1	87.1	0.001	1.3	ND	63.5	0.14	ND	0.38	0.61	603	1.18
11−49	94.3	8.1	14.0	45.1	374	57.9	3.4	0.002	0.5	ND	75.1	0.05	0.16	0.28	1.19	430	1.12
11−50	78.0	11.1	17.0	53.6	341	52.8	5.1	0.001	0.4	ND	74.2	ND	0.18	0.31	3.49	965	0.69
11−51	62.0	6.1	15.4	45.6	223	147.0	59.1	0.015	1.1	ND	78.0	ND	0.02	0.39	3.06	133	1.17
11−52	263.7	6.9	12.2	48.7	265	114.8	15.3	0.001	0.8	ND	86.9	ND	0.08	0.44	6.59	360	1.13
11−53	89.0	7.3	17.2	42.8	239	81.9	24.6	0.004	0.7	ND	41.1	ND	ND	0.25	1.96	107	1.05
11−54	125.6	8.8	18.0	48.6	239	86.5	13.2	0.005	0.6	ND	79.0	ND	0.16	0.29	1.70	193	1.09
11−55	30.3	7.3	18.9	46.4	521	56.6	16.5	0.005	0.6	ND	53.0	ND	2.21	0.31	2.98	194	0.77
11−56	61.0	4.5	10.0	26.0	147	88.1	9.6	0.002	0.5	ND	39.4	ND	0.02	0.22	4.57	41	1.25
11−57	178.1	12.6	16.8	55.6	194	105.2	78.0	0.005	0.5	ND	125.3	ND	0.09	0.26	3.18	257	0.95
11−59	86.4	8.9	18.2	41.0	453	68.6	17.0	0.004	0.3	ND	82.2	ND	0.34	0.25	1.22	163	0.97
11−61	166.4	3.3	7.8	38.9	322	83.9	3.1	0.005	0.4	ND	24.3	ND	0.19	0.12	2.00	152	0.80
11−62	24.8	3.9	9.9	26.8	156	43.9	5.6	0.001	0.4	ND	38.5	0.17	0.13	0.19	1.55	126	0.54
